# The amyloid precursor protein and its derived fragments concomitantly contribute to the alterations of mitochondrial transport machinery in Alzheimer’s disease

**DOI:** 10.1038/s41419-024-06742-2

**Published:** 2024-05-28

**Authors:** Loan Vaillant-Beuchot, Fanny Eysert, Blandine Duval, Paula Fernanda Kinoshita, Raphaëlle Pardossi-Piquard, Charlotte Bauer, Sabiha Eddarkaoui, Luc Buée, Frédéric Checler, Mounia Chami

**Affiliations:** 1 Université Côte d’Azur, INSERM, CNRS, Institute of Molecular and Cellular Pharmacology, Laboratory of excellence DistALZ, 06560 Sophia-Antipolis, Valbonne France; 2 Instituto de Ciências Biomédicas Department of Pharmacology, Universidade de São Paulo, São Paulo, Brazil; 3 Univ. Lille, Inserm, CHU-Lille, Lille Neuroscience and Cognition, Place de Verdun, 59045 Lille, France; 4 Inserm UMR-S 1172, Laboratory of excellence DistALZ, ‘Alzheimer and Tauopathies’, Bâtiment Biserte, rue Polonovski, 59045 Lille, Cedex France

**Keywords:** Alzheimer's disease, Mechanisms of disease

## Abstract

Mitochondria dysfunctions and mitophagy failure have been associated with several Alzheimer’s disease (AD) related molecular actors including amyloid beta (Aβ) and recently the amyloid precursor protein-C terminal fragments (APP-CTFs). The efficacy of the mitophagy process in neurons relies on regulated mitochondrial transport along axons involving a complex molecular machinery. The contribution of the amyloid precursor protein (APP) and its derived fragments to the mitochondrial transport machinery alterations in AD have not been investigated before. We report herein a change of the expression of mitochondrial transport proteins (SNPH and Miro1), motor adapters (TRANK1 and TRAK2), and components of the dynein and kinesin motors (i.e., IC1,2 and Kif5 (A, B, C) isoforms) by endogenous APP and by overexpression of APP carrying the familial Swedish mutation (APPswe). We show that APP-CTFs and Aβ concomitantly regulate the expression of a set of transport proteins as demonstrated in APPswe cells treated with β- and γ-secretase inhibitors and in cells Knock-down for presenilin 1 and 2. We further report the impact of APP-CTFs on the expression of transport proteins in AAV-injected C99 mice brains. Our data also indicate that both Aβ oligomers (Aβo) and APP-CTFs impair the colocalization of mitochondria and transport proteins. This has been demonstrated in differentiated SH-SY5Y naive cells treated with Aβo and in differentiated SH-SY5Y and murine primary neurons expressing APPswe and treated with the γ-secretase inhibitor. Importantly, we uncover that the expression of a set of transport proteins is modulated in a disease-dependent manner in 3xTgAD mice and in human sporadic AD brains. This study highlights molecular mechanisms underlying mitochondrial transport defects in AD that likely contribute to mitophagy failure and disease progression.

## Introduction

Mitochondria ensures several vital functions, including energy production through the synthesis of adenosine triphosphate (ATP) by the oxidative phosphorylation system [1]. Mitochondria organelles follow a dynamic transport via microtubules (MT) to fulfill their role locally in polarized cells such as neurons [2]. Thus, the anterograde transport occurring from the nucleus to the cell’s periphery (i.e., axons, dendrites, and synapses) distributes newly synthesized and healthy mitochondria, thereby ensuring the replenishment of these high energy demanding sites with ATP that is necessary for synaptic transmission and plasticity [3, 4]. In an opposite manner, dysfunctional mitochondria undergo a retrograde transport backward to the soma where they are degraded by mitophagy (i.e., the specific degradation systems of altered mitochondria) upon fusion of the mitophagosomes with lysosomes [5].

Mitochondrial transport along MT is achieved by a set of ATP-driven motors including kinesin for anterograde transport and cytoplasmic dynein for retrograde transport [6]. Mammalian kinesin isoforms include the neuronal Kif5A and Kif5C and the ubiquitous Kif5B [7]. Cytoplasmic dynein is a multi-subunit nanomotor composed of intermediate chains (ICs), light intermediate chains (LICs), and light chains (LCs) built around a core of large dimeric heavy chains (HCs), which perform ATP hydrolysis [8]. These motor proteins can carry several organelles and vesicles and interact with specific cargoes or cargo-adapter proteins to transport mitochondria [6]. Among motor adapters proteins, kinesin-binding proteins (TRAK1 and TRAK2) are implicated in the trafficking of different organelles, including endosomes, and interact indirectly with mitochondria through the mitochondrial rho (MIRO), an RHO family GTPase expressed at the mitochondrial outer membrane [9]. Miro1 isoform is the major mitochondrial acceptor in neurons [10]. The mechanisms linking dynein to mitochondria are not well characterized. However, it seems that MIRO may also be required for dynein-mediated retrograde mitochondrial transport [11]. Another protein that regulates mitochondrial transport is syntaphilin (SNPH) that blocks the global movement of mitochondria by directly anchoring the organelle on MT [4].

Alzheimer’s disease (AD) is a neurodegenerative disease initially defined by a proteinopathy associated with the accumulation of amyloid beta (Aβ) peptides that are produced through the sequential cleavage of the amyloid precursor protein (APP) by the β- and the γ-secretases [12]. These Aβ peptides accumulate extracellularly in senile plaques [13] and interact with different intracellular cellular components and organelles, including mitochondria [14, 15]. Besides Aβ, APP processing generates other fragments that have several toxic features contributing to the etiology of AD [16], namely the APP C-terminal fragments (APP-CTFs: C99 and C83). C99 fragment results from of the cleavage of APP by the β- secretase and C83 fragment derives from the cleavage of both APP and C99 by the α-secretase [17–20]. Accumulation of APP-CTFs in cellular models mimicking familial and sporadic forms of AD triggers an autolysosomal defect, which in turn impairs APP-CTFs degradation and induces their pathogenic accumulation [21, 22]. In a recent study, we reported that APP-CTFs accumulation is associated with mitochondrial structure and function alterations and triggers mitophagy failure in both cellular and mice AD models [23]. These findings are supported by a recent study demonstrating the accumulation of APP-CTFs in mitochondria and their causality of impaired mitophagy function in AD patient-induced neural stem cells (iNSCs) [24]. Importantly, APP-CTFs accumulation in sporadic human AD brains correlates with a mitophagy failure molecular signature and is associated with neurodegeneration [23, 25].

In parallel to mitochondria structure, function, and mitophagy alterations, isolated studies reported impaired mitochondrial transport in AD-related conditions [26]. However, the molecular mechanisms underlying mitochondrial transport defect in AD remain partially understood. Here, we investigated the implication of APP, APP-CTFs, and Aβ on the expression and localization of mitochondrial transport components (i.e., SNPH, Miro1, TRAK1 and 2, Kif5 A, B, C isoforms and IC 1, 2) using complementary cellular and mice AD models. We also studied the molecular signature of mitochondrial transport proteins associated with AD progression in post-mortem human brains.

## Materials and methods

### Human brain samples

We studied control and sporadic AD (SAD) post-mortem human brains (Control, *n* = 5; I–III, *n* = 5; and IV–VI, *n* = 14). Cases were anonymized, but information was provided regarding sex, age at death, and neuropathology (Table 1). Studies involving human brain samples were in accordance with the ethical standards of the institutional and/or national research committee and with the 1964 Helsinki Declaration and its later amendments or comparable ethical studies. Brain samples were obtained from the Brain Bank “NeuroCEB” run by a consortium of patient associations: ARSEP (association for research on multiple sclerosis), CSC (cerebellar ataxias), and France Parkinson. The consents were signed by the patients themselves or their next of kin in their name, in accordance with the French Bioethical (agreement AC-2013-1887).

**Table 1:** Demographic data and neuropathology evolution of human brain samples described by Braak neurofibrillary stages and Thal amyloid stages, used in SDS-PAGE.

	Thal stage	Braak stage	Gender	Age	PMD (h)	Brain area
CT	–	–	H	71	26	T1
CT	–	–	H	84	32	T1
CT	–	–	F	71	15	T1
CT	–	–	F	89	12	T1
CT	–	–	F	52	29	T1
AD	NA	I–II	F	68	51	T1
AD	0	II	F	83	21	T1
AD	2	II	M	85	10	T1
AD	0	III	F	92	NA	T1
AD	0	III	M	70	31	T1
AD	2	IV	F	76	28	T1
AD	NA	IV	M	92	NA	T1
AD	NA	V	M	71	NA	T1
AD	5	V	M	82	25	T1
AD	NA	V	F	80	51	T1
AD	0	VI	F	78	18	T1
AD	5	VI	F	65	41	T1
AD	4–5	VI	F	75	7	T1
AD	4	VI	F	89	26	T1
AD	NA	VI	F	93	21	T1
AD	5	VI	F	91	34	T1
AD^a^	5	VI	F	55	58	T1
AD	NA	VI	H	67	30	T1
AD	1	VI	F	82	NA	T1

Controls (CT) are brain samples isolated from post-mortem patients diagnosed as negative for several neuropathologies, we also used control post-mortem brain samples diagnosed as negative for AD pathology.

*NA* not available, *PMD* post-mortem delay in hours, *T1* region of the temporal lobe, *h* hours.

^a^Suspected to be a familial AD case.

### Animals

Experiments were performed on hippocampi dissected from 3xTgAD female mice (APPswe: KM670/671NL, TauP301L, and presenelin1 (PS1) M146V) (*n* = 8 aged 4 months and *n* = 7 aged 13 months) [27] and wild-type female mice (WT; non-transgenic) (*n* = 7 aged 4 months and *n* = 5 aged 13 months). We also used the brains of female mice aged 6 months and injected at day 1 post-natal with adeno associated virus (AAV)-C99 (*n* = 5) or AAV-Free (empty virus) (*n* = 4) [22]. Virus production of AAV10 expressing empty vector (AAV-Free) or the human APP C99 fragment (AAV-C99) under the control of the synapsin-1 promoter was performed following a protocol previously described [28]. For AAV-mediated in vivo delivery, 1-day-old C57BL6 mice (Janvier Labs., France) were injected with 4 µl of AAV virus (5.5 × 10^12^ vg/ml (viral genomes per ml)) into the left ventricle, as described [22]. Only left brain was used for protein extraction. Mice were housed with a 12:12 h light/dark cycle and were given free access to food and water.

### Ethical approval

Studies on animals were performed following the guidelines established by the European Community Council (Directive of November 24th, 1986), and applying ethical standards of the institution. Animal experimentation protocols were approved by the Nice University Animal Care and Use Committee, and the National Council on Animal Care of the French Ministry of Higher Education and Research (project #00253.02 and APAFIS#20495-201904231352370). These projects were evaluated and followed by the welfare animal use committee of the institute.

### Cells

We used human SH-SY5Y neuroblastoma cells (CRL-2266, ATCC) stably expressing empty pcDNA3.1 vector (Control), or the human βAPP harboring the double Swedish mutations cDNA (APPswe: APPKM670/671NL) [29]. We also used mouse embryonic fibroblasts: MEFs either control (MEF APPWT or MEF PSWT), invalidated for APP and for APP-like proteins 1 and 2 (APLP1 and APLP2) (MEF APPKO) [30], or doubly knocked out for presenilin 1 (PS1) and presenilin 2 (PS2) (PSDKO) [31]. Cells were maintained in Dulbecco’s modified Eagle’s medium (DMEM, GIBCO 41965-039) supplemented with 10% Fetal Bovine Serum (FBS), penicillin (100 U/ml) and streptomycin (50 μg/ml) (Pen/Strep), and incubated at 37 °C in 5% CO_2_ atmosphere. Control and APPswe SHSY5Y cells were cultured in the presence of 400 μg/ml geneticin (MP Biomedicals) [29].

### Treatments

Cells were treated with DMSO (Sigma D2650) as control (vehicle), γ-secretase inhibitor ELDN006 (3 nM in DMSO) [32] or β-secretase inhibitor LY2811376 (30 nM in DMSO) [33] for 16 h. Differentiated SHSY-5Y-APPswe were treated with γ-secretase inhibitor (0.3 nM) for 16 h to limit cell death.

### Differentiation of SHSY5Y cell lines

We used a protocol adapted from a previously reported protocol [34]. Briefly, cells were plated in a 6-well plate at a confluence of 30–40% to reach 70% maximum 5 days later. The first week of culture, we used M1 medium (DMEM supplemented with Pen/Strep and 2.5% of FBS, and 10 µM retinoic acid (RA, Merck R2526). Cells were then split into new 6-well plates and cultured in M2 medium (DMEM supplemented with Pen/Strep, 1% of FBS, and 10 µM RA). Cells were then split 2 days later in plates coated with 0.5 µg/ml Poly-L-lysin (Sigma–Aldrich P2636) in neurobasal medium complemented with B27 (1X), Pen/Strep, 50 ng/mL BNDF (Merck B3795), 2 mM Glutamax (Gibco 35050061), 20 mM KCL solution and 10 µM RA. Differentiated cells were used 3 weeks later.

### Primary cultures of murine neurons

Primary neuronal cultures were obtained from the hippocampus and cortices of mice at 16 days of embryonic stage. Isolated hippocampi and cortices were placed in a dissection medium (0.25% glucose, 1 mM Sodium pyruvate, 10 mM HEPES, HBSS), washed two times in HBSS, and dissociated by trypsin digestion (2.5% Trypsin, 0.025% BSA, 0.625% glucose in PBS) at 37 °C for 10 min. Hippocampi and cortices were then mechanically dissociated with DNAse (1 mg/mL, Sigma). Neurons were centrifugated at 200 × *g* for 5 min and the pellet was resuspended in Neurobasal medium (Gibco) supplemented with 2% B27 (Gibco), 1% GlutaMax (Gibco), and 1% Penicillin/Streptomycin (Gibco). Neurons were then filtered using 70 µm filter and seeded (50,000 per well) in 24-well plates on coverslips precoated with poly-L-lysine (0.05 mg/mL, Sigma). Neurons were maintained at 37 °C in a humidified 5% CO_2_ incubator.

### Transfection of primary neuronal cultures

Premature neurons were transfected at 7 days in vitro (DIV 7) with Mit-RFP [35] and pcDNA3.1 empty vector or APPswe construct [23] using Lipofectamine 2000 following the manufacturer’s instructions. At 14 DIV, neurons were treated with γ-secretase inhibitor at 5 µM for 4 h [23].

### Aβ_1–40_ and Aβ_1–42_ sandwich ELISA tests

The concentrations of human Aβ_1–40_ and Aβ_1–42_ were measured in total protein extract of human brains (temporal lobe area) by using the respective ELISA kits (Invitrogen) following the manufacturer’s instructions. Briefly, 50 µg of total protein extracts were dissolved in 5 M Guanidine HCl/ 50 mM Tris HCl, pH 8.0, to yield detection of both soluble and insoluble Aβ peptides.

### Immunofluorescence

Differentiated control or APPswe-expressing SH-SY5Y cells and primary neurons were fixed with 4% paraformaldehyde (PFA) for 10 min, washed twice with 1X PBS, and then permeabilized with 0.5% (cells) or 0.3% (neurons) Triton X-100 for 5 min. Non-specific binding sites were blocked for 1–2 h in 3% PBS-BSA containing 0.05% tween at room temperature. Primary antibodies were diluted (Supplementary Table 1) in PBS-BSA 0.3% containing 0.005% tween and applied overnight (ON). After three washes with 1xPBS, AlexaFluor-conjugated secondary antibodies (Jackson ImmunoResearch) were applied for 1 h. Nuclei were stained with DAPI (1/10000, Invitrogen) and slides were mounted with Vectamount medium (Vector). Images were taken with a confocal Leica TCS SP5 microscope or LSM 780. The colocalization quantification was determined using the Fiji plug-in JACoP (Just Another Colocalization Plug-in).

### Protein extraction and SDS-PAGE and western blot analysis

Total protein extracts were obtained from cells, dissected mice hippocampi of WT and 3xTgAD, cortices of AAV-Free or AAV-C99 mice, or human temporal cortex using lysis buffer (50 mM Tris pH 8, 10% glycerol, 200 mM NaCl, 0.5% Nonidet p-40, and 0.1 mM EDTA) supplemented with protease inhibitors (Complete cocktail, Roche diagnostics 04693132001). Proteins were resolved by SDS-PAGE following standard procedures and APP-CTFs were resolved on 16.5% Tris-Tricine SDS-PAGE [23]. Information about the primary antibodies used for western blot can be found in Supplement Table 1.

### Statistical analyses

Data were expressed as means ± SEM. Sample size for each experiment is indicated in the Figure captions and data are presented in bar blots and scatter plots. The data are averaged from technical replicates in each independent experiment. The quantification of imaging experiments was performed on different (>5) fields of view obtained from independent experiments. Data were analyzed with GraphPad Prism version 9 for Windows (GraphPad Software, La Jolla, CA, USA; https://www.graphpad.com). Experiments were not blinded. However, they were conducted and replicated by different authors. Data were first analyzed for normal distribution. We used the Mann–Whitney test when the two groups of variables had not passed the normality test. Groups of more than two variables that have passed the normality test were analyzed by one-way ANOVA with Dunnett’s or Tukey’s multiple comparisons post-test. Kruskal–Wallis test and Dunn’s multiple comparisons post-test were used when groups of variables have not passed the normality test. Correlations were analyzed with non-parametric Spearman’s correlation. We compute correlation between every pair of data sets and when a value is missing, we remove the entire row from the calculation. Significant differences are: *P < 0.05, **P < 0.01, ***P < 0.001, ****P < 0.0001 and ns non-significant.

## Results

### APP and its derived fragments modulate the expression of proteins of the mitochondrial transport machinery

**Fig. 1:**
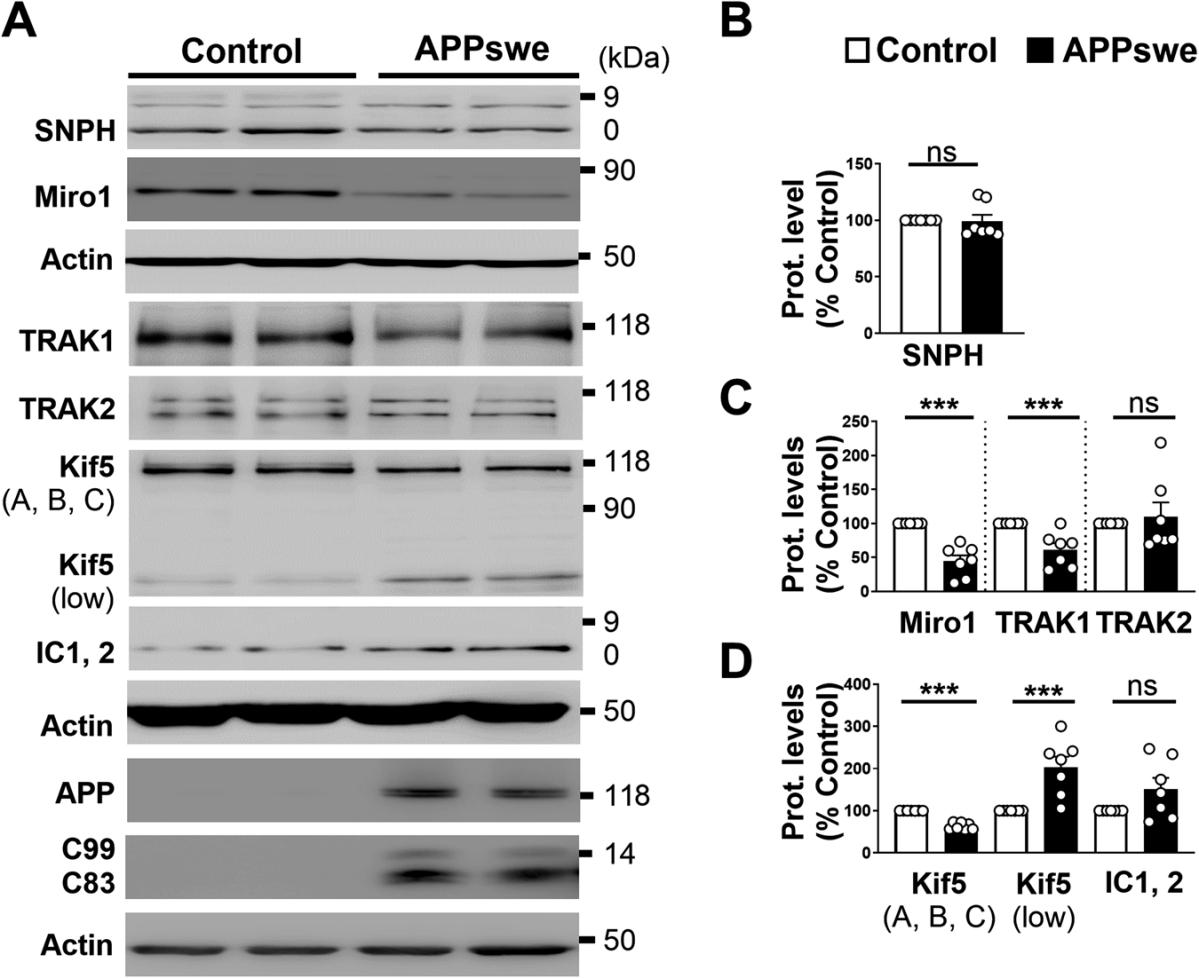
SHSY-5Y cells expressing APPswe show altered expression levels of mitochondrial transport proteins. **A** SDS-PAGE of SNPH, Miro1, TRAK1, TRAK2, Kif5 (A, B, and C), IC 1, 2 in SHSY-5Y cells stably transfected with APPswe or pcDNA3.1 used as control. Kif5 (low) is an additional band recognized by Kif5 antibody. Full-length APP and APP-CTFs were revealed in Tris-Tricine SDS-PAGE. Representative SDS PAGE of β-actin is shown as loading controls. Full-length western blots are provided in supplementary data. Quantitative graphs of SNPH (**B**), Miro1, TRAK1, TRAK2 (**C**), and Kif5 (A, B, C), Kif5 (low), and IC1, 2 (**D**) protein levels expressed as means ± SEM of control (set at 100%). Data were obtained from seven independent experiments. *** *P* < 0.001, and ns non-significant versus control using Mann–Whitney’s test.

We studied the expression of the mitochondrial stop protein syntaphilin (SNPH), the mitochondrial rho protein (Miro1), the kinesin-binding proteins (TRAK1 and TRAK2), the motor kinesin (Kif5 (A, B, C) isoforms), and the ICs of the cytoplasmic dynein motor (lC1,2) in neuroblastoma cells expressing APP carrying the double Swedish familial mutations KM670/671NL (APPswe) and their control cells expressing pcDNA3.1 empty vector (Control) [29] (Fig. 1). This cellular model has been previously described to accumulate APP-derived fragments (i.e., Aβ and APP-CTFs) and to manifest mitochondrial structure and function alterations and a defect of mitophagy process [23]. We noticed a reduction in the expression of Miro1 and TRAK1 (Fig. 1A, C), and Kif5 (A, B, C) (Fig. 1A, D) in APPswe versus control cells, while the expression of SNPH (Fig. 1A) and TRAK2 (Fig. 1C) remained unchanged (Fig. 1A, C) and that of IC1, 2 level is slightly but not significantly increased (Fig. 1A, D). We identified a low molecular weight (≈50 kDa) second band recognized by the Kif5 (A, B, C) antibody that we labeled as Kif5 (low) showing in APPswe cells a mirror upregulation versus full-length Kif5 (A, B, C) (Fig. 1A, D).

**Fig. 2:**
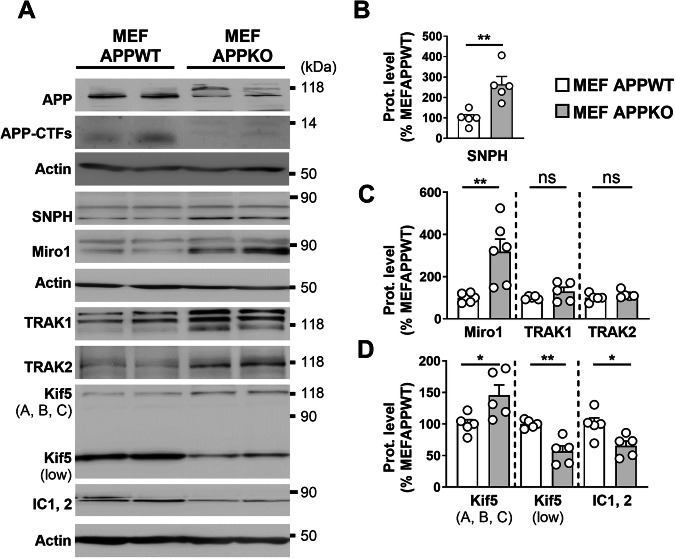
MEF APPDKO shows altered expression levels of mitochondrial transport proteins. **A** SDS-PAGE of APP, APP-CTFs, SNPH, Miro1, TRAK1, TRAK2, Kif5 (A, B, C), and IC1, 2 in MEF APPWT and MEF APPKO. representative SDS PAGE of β-actin are shown as loading controls. Full-length western blots are provided in supplementary data. Quantitative graphs of SNPH (**B**), Miro1, TRAK1, TRAK2 (**C**), and Kif5 (A, B, C), Kif5 (low), and IC1, 2 (**D**) protein levels expressed as means ± SEM of MEF APPWT (set at 100%). Data were obtained from 5–6 independent experiments. *P < 0.05, **P < 0.01, and ns non-significant versus MEF APPWT using Mann–Whitney’s test.

Because APP is involved in several housekeeping cellular functions [36], we investigated whether the down expression of full-length wild-type APP may impact the expression of mitochondrial transport proteins. Since APP shares some functional redundancies with the amyloid precursor-like proteins APLP1 and APLP2, we compared control mouse embryonic fibroblasts (MEF APPWT) with cells depleted of APP and of APP-like proteins 1 and 2 (APLP1 and APLP2) (MEF APPKO). We confirmed reduced APP expression and observed the absence of endogenous APP-CTFs levels in MEFs APPKO cells (Fig. 2A). We evidenced an enhanced expression of SNPH, Miro1, and Kif5 (A, B, C) in MEFs APPKO cells when compared to controls (Fig. 2A–D) and noticed a mirror downregulation of Kif5 (low) band versus full-length Kif5 protein and of IC1, 2 protein in MEFs APPKO (Fig. 2A, D).

Neuroblastoma cells show a “compacted dense shape” of the mitochondria reticulum that was technically not adapted for the analyses of mitochondrial movement using imagery and time-laps acquisition. Thus, we studied mitochondria movement in MEF presenting “a flat shape mitochondria reticulum” and we revealed a reduced mitochondrial movement in MEF APPKO compared to MEF APPWT (Supplementary Video. 1, and Supplementary Fig. 1A). Since the expression levels of several proteins implicated in mitochondria movement are upregulated in MEF APPKO, we speculate that the reduction of mitochondria motile fraction in APPKO cells is associated with enhanced expression of SNPH known to block mitochondria movement (Fig. 2A, B).

Together, these pieces of data point out a contribution of endogenous APP and of APP-derived fragments accumulation in the control of the expression of a set of proteins constituting the mitochondrial transport machinery complex.

### Accumulation of both Aβ and APP-CTFs modulates the expression of several proteins of the mitochondrial transport machinery complex

**Fig. 3:**
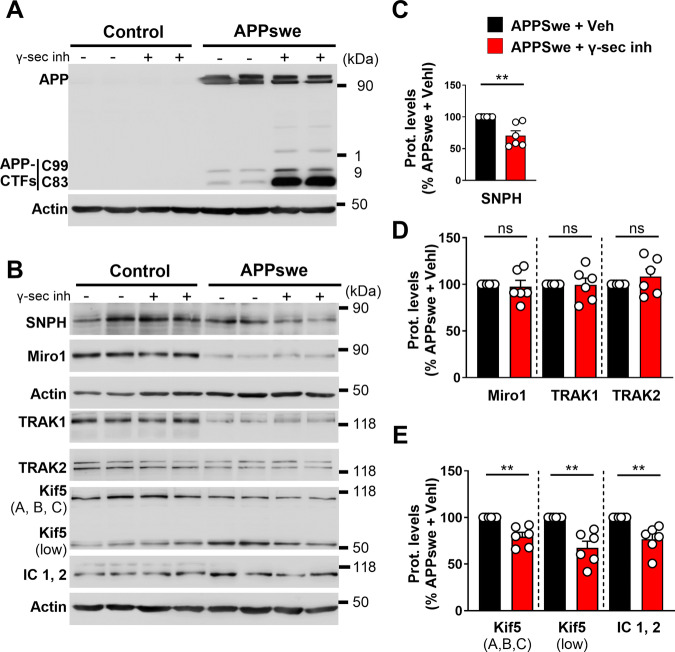
APP-CTFs accumulation in SHSY-5Y cells expressing APPswe induces specific modulation of the expression of mitochondrial transport proteins. **A** SDS-PAGE of full-length APP and APP-CTFs (C99, C83) levels in control and APPswe cells treated with vehicle (Veh) or with γ-secretase inhibitor (γ-sec inh). **B** SDS-PAGE of SNPH, Miro1, TRAK1, TRAK2, Kif5 (A, B, and C), Kif5 (low), and IC1, 2 in control and APPswe cells treated with vehicle or γ-secretase inhibitor. Representative SDS PAGE of β-actin is shown as loading controls. Full-length western blots are provided in Supplementary data. Quantitative graphs of SNPH (**C**), Miro1 and TRAK1 and TRAK2 (**D**), and Kif5 (A, B, C), Kif5 (low), and IC1, 2 (**E**) protein levels expressed as means ± SEM of APPswe + Veh (set at 100%). Data were obtained from six independent experiments. **P < 0.01 and ns non-significant versus control or APPswe + Veh using Kruskal–Wallis test and Dunn’s multiple comparison post-test.

In order to discriminate between the impact of Aβ and APP-CTFs on the expression of transport proteins, we used: i) the β- and the γ-secretases inhibitors in APPswe cells, to modulate the processing of APP and the levels of APP-CTFs (C99 and C83) and Aβ; and ii) MEF double knock-out for presenilins 1 and 2 (components of the γ-secretase complex) (MEF PSDKO). We previously reported that both β- and γ-secretases inhibitors block Aβ production in APPswe cells [37]. We also performed a new set of experiments and confirmed that, while the inhibition of the γ-secretase triggers an accumulation of both C99 and C83 fragments in APPswe cells (Fig. 3A, and Supplementary Fig. 2A), the inhibition of the β-secretase blocks C99 production and enhances the level of C83 (Supplementary Fig. 2A). We then showed that γ-secretase inhibition in APPswe cells triggers a reduction in SNPH levels (Fig. 3B, C) and potentiates the reduction in the expression of KIF5 (A, B, C) (Fig. 3B, E). In parallel, we observed that the reduced expression in Miro1 and TRAK1 in APPswe cells remained unchanged upon γ-secretase inhibition (Fig. 3B, D), suggesting the prevalence of APP-CTFs accumulation over Aβ blockade in these alterations. Finally, we showed that the inhibition of the γ-secretase abolishes the APP-induced increases in Kif5 (low) and IC1,2 (Fig. 2B, E), thus signifying a potential implication of Aβ accumulation in these alterations. These observations indicate that the expression levels of mitochondrial transport proteins are mitigated by either APP-CTFs or Aβ accumulation.

In the same sets of experiments, we assessed the impact of γ-secretase inhibition in control cells and noticed that the endogenous accumulation of APP-CTFs [23] does not impact the expression of mitochondrial transport proteins (Supplementary Fig. 3 A–C). We also reported that β-secretase inhibition in APPswe cells does not impact the expression of our proteins of interest (Supplementary Fig. 2B), indicating the prevalence of C99 over C83 in the observed effects upon γ-secretase inhibition.

Since γ-secretase has over 150 substrates [38], we undertook to ascertain that the changes we observed in APPswe cells are genuinely linked to APP-CTFs over accumulation and not to other γ-secretase substrates. We thus demonstrated that the reported modulations of mitochondrial transport proteins (Fig. 2) are not observed in MEF APPKO treated with the γ-secretase inhibitor (Supplementary Fig. 4). This confirmed that the modulation of protein expressions observed in APPswe cells was not due to the processing of other substrates than APP by γ-secretase.

**Fig. 4:**
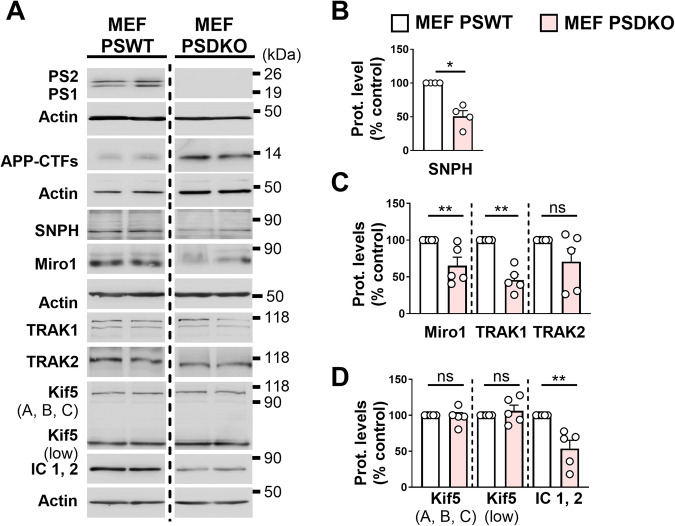
MEF PSDKO shows altered expression levels of mitochondrial transport proteins. **A** SDS-PAGE of PS1, PS2, APP-CTFS, SNPH, Miro1, TRAK1 and TRAK2, Kif5 (A, B, C), IC 1, 2 in MEFs PSWT and PSDKO. Representative SDS PAGE of β-actin is shown as loading controls. MEF WT and MEF PSDKO samples were loaded in the same gels. The dashed line indicates that unrelated samples loaded between MEF WT and MEF PSDKO samples are not shown. Full-length western blots are provided in supplementary data. Quantitative graphs of SNPH (**B**), Miro1, TRAK1, TRAK2 (**C**), and Kif5 (A, B, C), Kif5 (low), and IC1, 2 (**D**) protein levels expressed as means ± SEM of MEF WT (set at 100%). Data were obtained from 4–5 independent experiments. *P < 0.05, **P < 0.01, and ns non-significant versus MEF PSWT using Mann–Whitney’s test.

In addition, we studied the expression of the mitochondrial transport machinery complex in MEF PSDKO in which an accumulation of APP-CTFs occurs (Fig. 4A). Interestingly, MEF PSDKO showed a significant reduction in SNPH, Miro1, TRAK1, and IC1,2 levels when compared to control MEF (MEFs PSWT) (Fig. 4A–D), thus almost mimicking the results obtained in APPswe cells treated with the γ-secretase inhibitor and supporting a contribution of APP-CTFs accumulation to the alteration of the expression of mitochondrial transport machinery.

Intriguingly, we noticed that the expression of full-length Kif5 (A, B, C) and its lower band were not altered in MEF PSDKO (Fig. 4A, D), while they were regulated in APPswe-cells treated with the γ-secretase inhibitor. This may suggest that Kif5 low band production is regulated in a cell-type specific manner (SH-SY5Y versus MEFs) and/or does not solely implicate APP-CTFs accumulation.

All together, these data point out a drastic deregulation of mitochondrial transport proteins linked to PS knock-down. In fact, we revealed a reduced mitochondrial movement in MEF PSDKO when compared to their controls (Supplementary Video 2 and Supplementary Fig. 1B).

Thus, both genetic depletion and pharmacological blockade of γ-secretase concur to support a major deleterious effect of APP-CTFs accumulation on the expression of mitochondrial transport machinery.

### APP and some of its derived fragments alter the colocalization of several proteins of the mitochondrial transport machinery complex with mitochondria

**Fig. 5:**
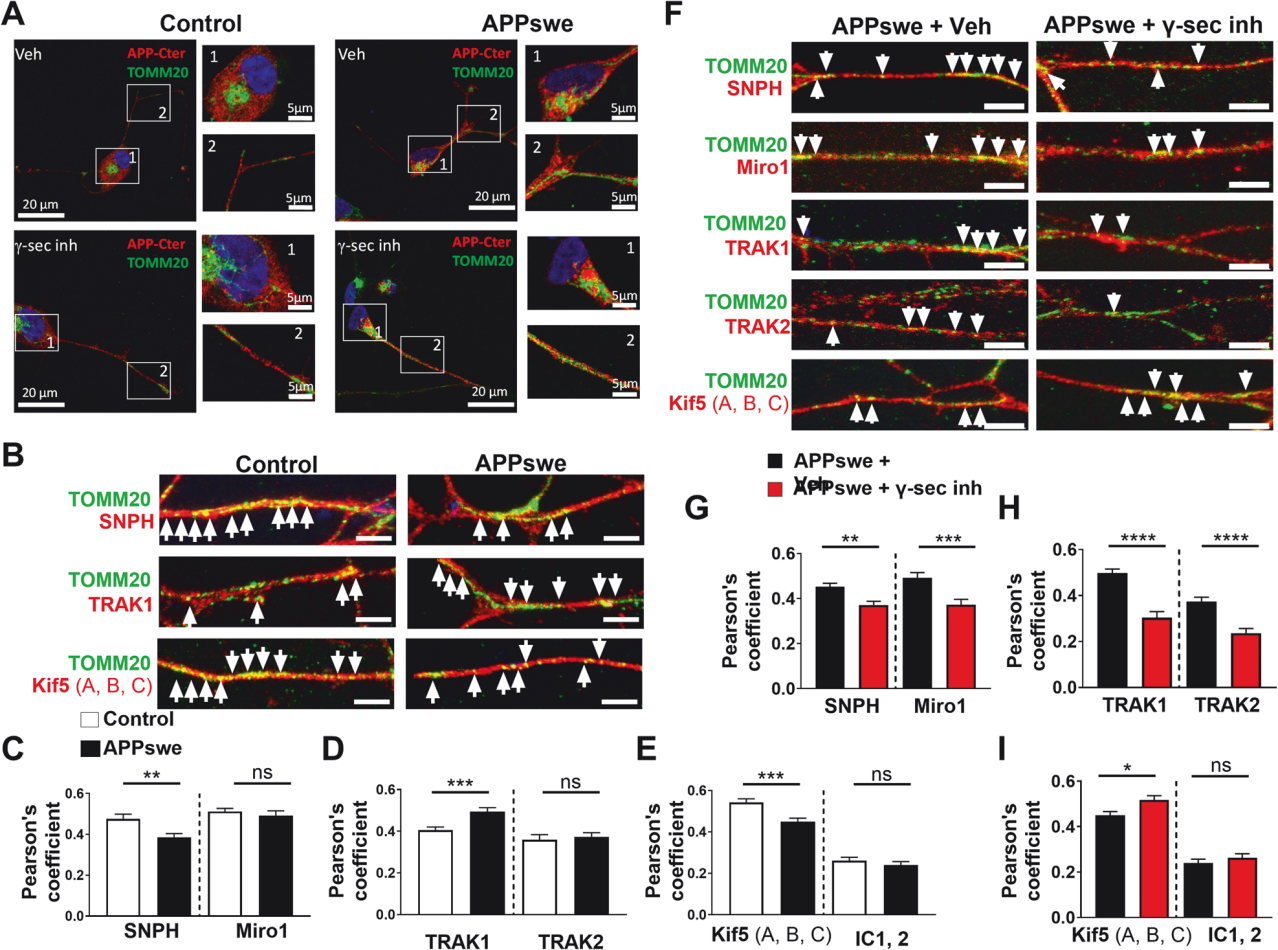
Mitochondria colocalization with transport proteins is affected in differentiated SH-SY5Y APPswe cells and with APP-CTFs accumulation. **A** Differentiated SH-SY5Y APPswe cells provide a complementary AD “neuronal-like” study model. Representative Immunofluorescence images showing the expression of APP and its C- terminal derived fragments detected with APP-Cter antibody (red) and mitochondria stained with TOMM20 antibody (green) in control and APPswe differentiated cells treated with vehicle (veh) or with γ-secretase inhibitor (γ-sec inh). The co-localization of APP and APP-CTFs with mitochondria is depicted in yellow (merge images). Nuclei are stained with Dapi. Scale bars = 20 µm or 5 µm. High magnificence of the soma (1) and dendrites (2). **B** Representative immunofluorescence images showing the colocalization of transport proteins (SNPH, TRAK1, and Kif5 in red) with mitochondria (TOMM20 in green) in differentiated control and APPswe SH-SY5Y cells. Scale bar = 5 µm. Quantitative graphs of the Pearson’s coefficient representing the colocalization (arrows) of SNPH and Miro1 (**C**), TRAK1 and TRAK2 (**D**), and Kif5 and IC1, 2 (**E**) with mitochondria. **F** Representative immunofluorescence images showing the colocalization of transport proteins (SNPH, Miro1, TRAK1, TRAK2, and Kif5 in red) with mitochondria (TOMM20 in green) in differentiated APPswe SH-SY5Y cells treated with vehicle (Veh) or with γ-secretase inhibitor (γ-sec inh) Scale bar = 5 µm. Quantitative graphs of the Pearson’s coefficient representing the colocalization of SNPH and Miro1 (**G**), TRAK1 and TRAK2 (H), and Kif5 and IC1, 2 (**I**) with mitochondria. **C**–**E** and **G**–**I** Graphs are expressed as means ± SEM. Data were obtained from four independent experiments. *P < 0.05, **P < 0.01, ***P < 0.001, ****P < 0.0001, and ns non-significant versus control using Mann–Whitney’s test.

We used differentiated SH-SY5Y cellular models to investigate the localization of proteins of the mitochondrial transport machinery complex in a polarized cellular context. We first notice a reduction of the proliferation of control and APPswe cells and validate their morphological changes towards a phenotype of polarized cells approaching that of neurons (Supplementary Fig. 5). Differentiated control and APPswe SH-SY5Y cells show branched morphology as revealed by an increase of the staining with two microtubule markers (β3-tubulin and microtubule-associated proteins, MAP2) (Supplementary Fig. 5). Differentiated cells also show an increase of the staining of neuronal marker NeuN and of tyrosine hydroxylase (a marker for dopaminergic and adrenergic neurons) (Supplementary Fig. 5). We also verified that differentiated APPswe cells provides an AD “neuronal-like” study model showing an evident colocalization of APP-CTFs with mitochondria (γ-secretase inhibitor treatment of APPswe) in both the soma and dendrites (Fig. 5A).

**Fig. 6:**
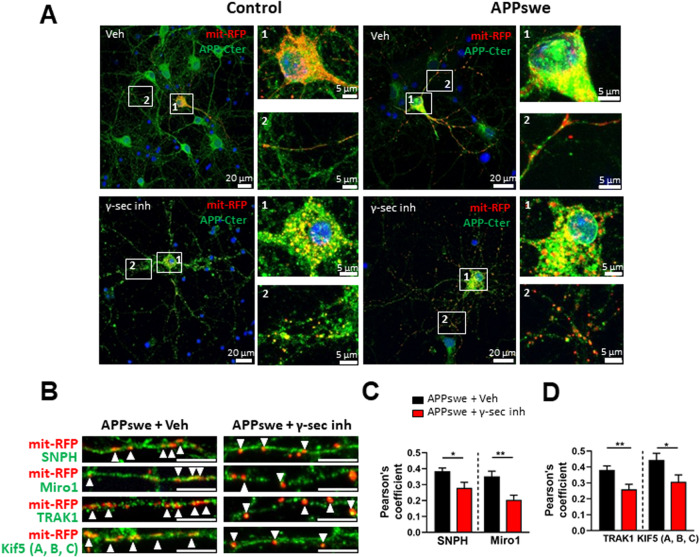
Mitochondria colocalization with transport proteins is affected in primary neurons expressing APPswe and accumulating APP-CTFs. **A** Primary neuron was co-transfected with mi-RFP probe and empty pcDNA3.1 (Control) or APPswe constructs. Representative immunofluorescence images showing the expression of APP and its C-terminal derived fragments detected with APP-Cter antibody (green) and mitochondria depicted with mit-RFP probe (red). Control and APPswe transfected primary neurons were treated with vehicle (veh) or with γ-secretase inhibitor (γ-sec inh). The co-localization of APP and APP-CTFs with mitochondria is shown in yellow (merge images). Nuclei are stained with Dapi. Scale bars = 20 µm or 5 µm. High magnificence of the soma (1) and dendrites (2). **B** Representative immunofluorescence images showing the colocalization of transport proteins (SNPH, Miro1, TRAK1, and Kif5 in green) with mit-RFP in primary neurons expressing APPswe treated with vehicle (veh) or with γ-secretase inhibitor (γ-sec inh). Scale bar = 5 µm. Quantitative graphs of the Pearson’s coefficient representing the colocalization (arrows) of SNPH and Miro1 (**C**), and TRAK1 and Kif5 (**D**) with mit-RFP. Graphs are expressed as means ± SEM. Data were obtained from two independent experiments and in two replicates per experiment (*n* > 15 images/condition). *P < 0.05, **P < 0.01 versus APPswe + Veh condition using Mann–Whitney’s test.

We then determined the intracellular localization of SNPH, Miro1, TRAK1, TRAK2, Kif5, and IC1,2 by immunofluorescence and we stained mitochondria using TOMM20 antibody. Differentiated APPswe cells show a reduction of the localization of SNPH (Fig. 5B, C), and Kif5 (Fig. 5B, E) in mitochondria, and an increase of the localization of TRAK1 (Fig. 5B, D) in mitochondria, while the localization of Miro1, TRAK2, and IC1,2 remained unchanged (Fig. 5C–E). Interestingly, γ-secretase inhibition amplifies this phenomenon by triggering an additional diminution of the mitochondrial localization of SNPH and Miro1 (Fig. 5F, G), and of TRAK1 and TRAK2 (Fig. 5F, H). These data demonstrate that the localization of a set of transport machinery components to mitochondria is impacted by APP-CTFs accumulation. Noticeably, we observed an increase in the localization of Kif5 in mitochondria upon γ-secretase inhibition (Fig. 5F, I), while the localization of IC1,2 remained unchanged (Fig. 5I). It should be mentioned here that Kif5 (A, B, C) antibody used in the immunofluorescence analyses cannot discriminate between full-length Kif5 or cleaved Kif5 (low) forms as detected by SDS-PAGE (Figs. 1–4). We then thought to validate these observations in primary murine neurons co-transfected with APPswe construct and mit-RFP probe (Fig. 6A). We first report enhanced localization of APP with Mit-RFP probe in APPswe expressing neurons that are exacerbated upon γ-secretase inhibition as compared to control neurons expressing the empty vector. As in differentiated neuroblastoma cells, the colocalization of APP-CTFs with mitochondria was observed in both the soma and dendrites. We also evidenced a fragmentation of mitochondria structure upon γ-secretase inhibition in both control and APPswe-expressing neurons (Fig. 6A, inset 2). Importantly, we observe in primary neurons, that APP-CTFs accumulation triggered by the γ-secretase inhibition induces a reduction of the mitochondrial localization of SNPH and Miro1 (Fig. 6B, C), and of TRAK1 and Kif5 (A, B, C) (Fig. 6B, D). Noticeably, only Kif5 (A, B, C) show opposite results between differentiated neuroblastoma (increased localization with mitochondria) and primary neurons (reduced localization with mitochondria), likely linked to stable versus transient expression of APPswe in both models. Intriguingly, while the expression of transport proteins was almost not altered in APPswe cells treated with β-secretase inhibitor (Supplementary Fig. 2), we noticed in these cells a reduction in the localization of Miro1, TRAK 1 and TRAK2 (Supplementary Fig. 6A–C) and a slight increase in IC1, 2 localization to the mitochondria (Supplementary Figs. 6A, D). These data support a contribution of the α-secretase-derived APP fragment (C83) accumulation to the alteration of the localization of some transport machinery components to mitochondria.

In a complementary set of experiments, we investigated the specific impact of Aβ oligomers (Aβo) on the localization of the same set of transport proteins in mitochondria using control differentiated SH-SY5Y cells. Aβo treatment triggers a reduction in the localization of SNPH, Miro1 (Supplementary Fig. 7A, B), and TRAK1 (Supplementary Fig. 7A, C) to mitochondria, while the localization of TRAK2, Kif5, and IC1, 2 remains unchanged (Supplementary Fig. 7A, C and D).

Together, these data (Figs. 5 and 6 and Supplementary Figs. 6 and 7) firmly demonstrate a defect of the mitochondrial transport machinery localization to mitochondria linked to Aβ and APP-CTFs (C99 and C83).

### The expression of several proteins of the mitochondrial transport machinery complex is altered in 3xTgAD and AAV-C99 mice

**Fig. 7:**
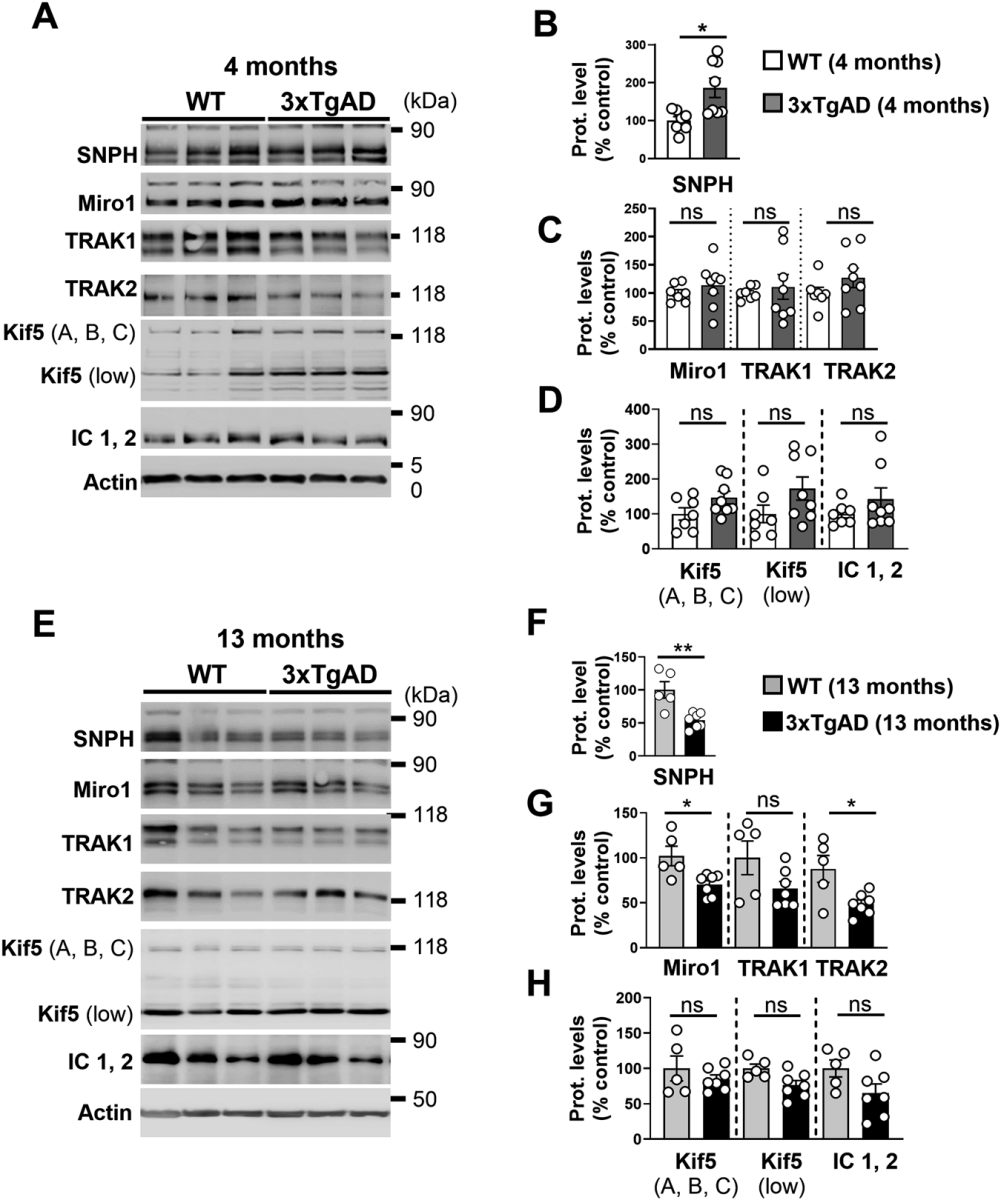
3xTgAD mice show an age-dependent modulation of the expression SNPH, Miro1 and TRAK2. SDS-PAGE of SNPH, Miro1, TRAK1, TRAK2, Kif5 (A, B, C), Kif5 (low), and IC1, 2 in control wild type (WT) and 3xTgAD mice aged 4 months old (**A**) or 13 months old (**E**). Representative SDS PAGE of β-actin is shown as loading controls. Full-length western blots are provided in supplementary data. Quantitative graphs of SNPH (**B**, **F**), Miro1, TRAK1, and TRAK2 (**C**, **G**), and Kif5 (A, B, C), Kif5 (low), and IC1, 2 (**D**, **H**) protein levels expressed as means ± SEM of WT mice (set at 100%). Data were obtained from 3xTgAD mice (*n* = 8 aged 4 months and *n* = 7 aged 13 months) and WT mice (*n* = 7 aged 4 months and *n* = 5 aged 13 months). * *P* < 0.05, ** *P* < 0.01, and ns non-significant versus WT mice using Mann–Whitney’s test.

**Fig. 8:**
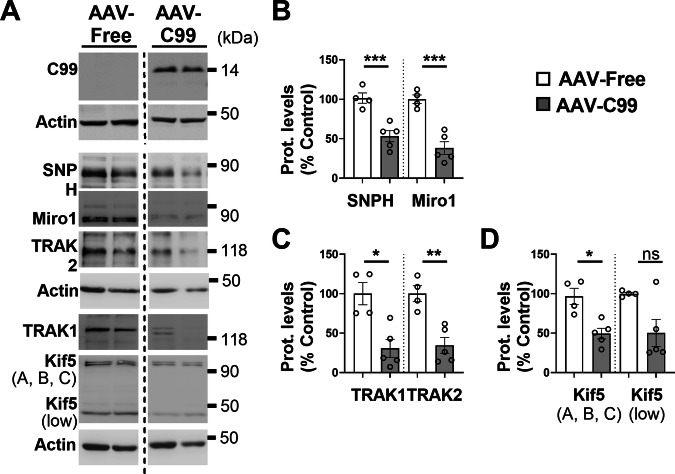
AAV-C99 injected mice show modulations of the expression of mitochondrial transport machinery proteins. **A** SDS-PAGE of C99 and of SNPH, Miro1, TRAK1, TRAK2, Kif5 (A, B, C), and Kif5 (low) in control (AAV-Free) and AAV-C99 injected mice aged 6 month-old. Representative SDS PAGE of β-actin is shown as loading controls. AAV-Free and AAV-C99 samples were loaded in the same gel. The dashed line indicates that samples loaded between AAV-Free and AAV-C99 samples are not shown. Full-length western blots are provided in supplementary data. Quantitative graphs of SNPH and Miro1 (**B**), TRAK1 and TRAK2 (**C**), and Kif5 (A, B, C) and Kif5 (low) (**D**) protein levels expressed as means ± SEM of AAV-Free mice (set at 100%). Data were obtained from AAV-C99 (*n* = 5) and AAV-Free (*n* = 4). *P < 0.05, **P < 0.01, and ***P < 0.001 and ns non-significant versus AAV-free mice using Mann–Whitney’s test.

We then studied the expression of our proteins of interest in the hippocampus of the 3xTgAD mice that harbor an early and progressive accumulation of APP-CTFs starting at 2–3 months of age followed by the production and the accumulation of Aβ at 6 months of age and forming the amyloid plaques at 10–11 months of age [18, 27, 39]. We first revealed an increase in SNPH expression in young 3xTgAD mice (aged 4 months) hippocampi when compared to age-matched control mice hippocampi (Fig. 7A, B), while the levels of the expression of Miro1, TRAK1, TRAK2, Kif5, and IC1,2 expressions remain unchanged or slightly but not significantly increased (Fig. 7A, C, and D). On the contrary, we reported a significant decrease in SNPH, Miro1, and TRAK2 in old 3xTgAD mice (aged 13 months) when compared to age-matched control mice (Fig. 7E, F, and G). In parallel, while we noticed a trend decrease in the expression of TRAK1 and IC1,2 in old 3xTgAD mice hippocampi, Kif5 (A, B, C) and Kif5 (low) remain unchanged (Fig. 7A, G, and H). In addition to APPswe mutation, the 3xTgAD mice overexpress a mutated form of the Tau protein (TauP301L) and are knocked in for presenilin1 (PS1) carrying M146V mutation. Thus, in addition to an impact of APP-derived fragments, we cannot exclude a potential contribution of mutated PS1 and/or phosphorylated Tau to the alterations of the expression of the mitochondrial transport machinery complex observed in this model. To investigate the specific impact of the C99 accumulation on mitochondrial transport machinery in vivo, we used AAV-C99 mice overexpressing C99 fragment in an endogenous APP, PS, and Tau background [23]. Interestingly, SDS-PAGE analyses revealed a drastic reduction in the expression of SNPH, Miro1, TRAK1, TRAK2, and Kif5 (A, B, C) in AAV-C99 injected mice brains when compared to AAV-Free injected controls (Fig. 8A–D), thus revealing a role of APP-CTFs accumulation in mitochondrial transport alteration in vivo.

### The expression of a set proteins of the mitochondrial transport machinery complex is altered in sporadic AD human brains

**Fig. 9:**
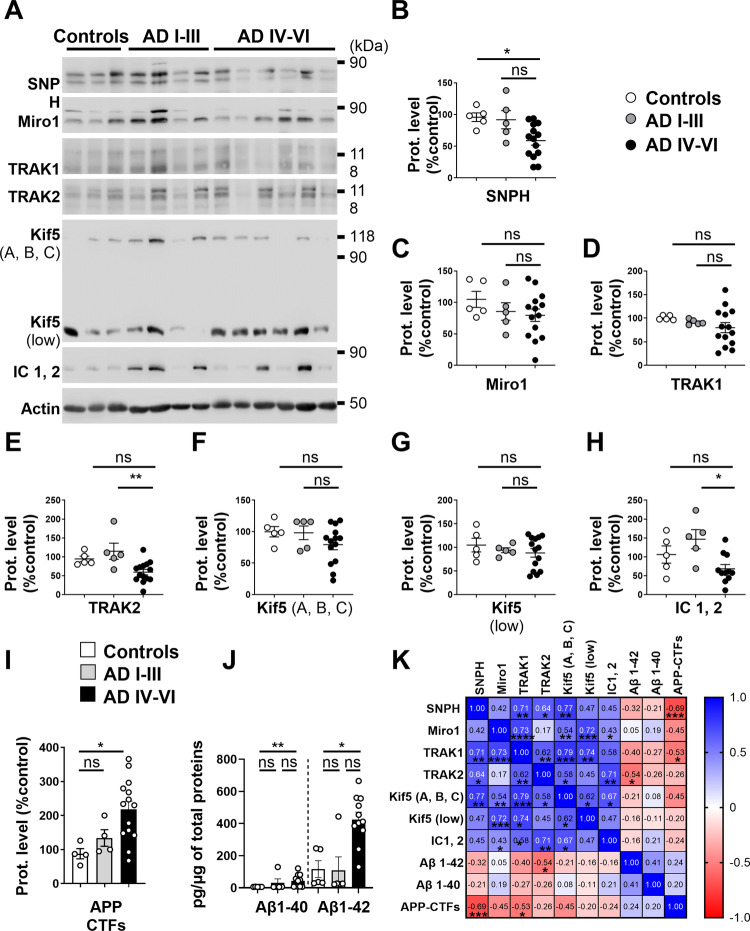
Sporadic human AD brains at late Braak stages show modulations of the expression of SNPH, TRAK2, and IC1, 2 proteins. **A** SDS-PAGE of SNPH, Miro1, TRAK1, TRAK2, Kif5 (A, B, C), Kif5 (low), and IC 1,2. Quantitative data were stratified in three categories: Controls including post-mortem brains of non-demented individuals, Early stages of Braak AD brains (AD I–III), and late stages of Braak AD brains (AD IV–VI). Representative SDS PAGE of β-actin is shown as loading control. Full-length western blots are provided in supplementary data. **B**–**H** Quantitative dot plots and graphs of indicated proteins expressed as means ± SEM versus controls (taken as 100%). **I** Quantitative dot plots and graphs of APP-CTFs levels as means ± SEM versus controls (taken as 100%). **J** Quantitative dot plots and graphs of Aβ1-40 and Aβ&-42 levels (pg/µg of total brain proteins) Data were obtained from Control (*n* = 4–5), AD I–III (*n* = 4–5) and AD IV–VI (*n* = 11–14). **K** Heat map of the correlation matrix computing Spearman *r* value for every pair of data sets is displayed in each box. Color scale of Spearman r values where 1 represents the maximum positive correlation value (blue) −1 represents the maximum negative correlation value (red), and 0 represents no correlation (white). **B**–**J** *P < 0.05, **P < 0.01, and ns non-significant versus Control or AD I-III using Kruskal–Wallis test and Dunn’s multiple comparison post-test. *K*, *P* values for the significance between modules *P < 0.05, **P < 0.01, ***P < 0.001, ****P < 0.0001.

We lastly questioned whether human SAD brains manifest an altered expression of mitochondrial transport proteins. We stratified AD brains into early Braak stages (I-III) and late Braak stages (IV–VI) (patients’ information in Table 1). Quantitative analyses revealed a significant reduction in the expression of SNPH in Braak IV-VI AD brains versus age-matched non-demented control brains (Fig. 9A, B), while TRAK2 (Fig. 9A, E) and IC1,2 (Fig. 9A, H) levels were gradually reduced in a disease-dependent manner (i.e., Braak IV–VI AD brains versus Braak stages (I–III) AD brains). Interestingly, correlation studies including control and AD brains revealed a statistically significant positive correlation between the expression levels of several transport proteins (Fig. 9K), namely between SNPH and TRAK1, TRAK2, and Kif5 (A, B, C). Miro1 expression also positively correlates with that of TRAK1, Kif5 (A, B, C), Kif5 low, and IC1, 2. Both TRAK1 and TRAK2 expressions correlate with Kif5 (A, B, C). Finally, IC1, 2 expression level correlates with TRAK2 and Kif5 (A, B, C) (Fig. 9K). These correlations have to be put in perspective with the fact that transport proteins follow a decreased expression trend in a disease-dependent manner in AD. We further documented the relationship between the level of APP-CTFs and Aβ (Fig. 9I-K and Supplementary Fig. 8A), and the expression of transport proteins and showed statistically significant negative correlations of SNPH, and TRAK1 levels with APP-CTFs levels, and of TRAK2 expressions with Aβ level (Fig. 9K). These AD brains also manifest enhanced phosphorylation of Tau on threonine231(pTauT231) and serine422 (pTauS422) residues quantified versus total Tau protein (tTau) (Supplementary Fig. 8B). However, we did not find significant correlations between the expression of transport proteins and pTauS422 but not with pTauT231 levels (Supplementary Fig. 8C).

Together, these data support the findings in cellular and mice AD models mimicking familial forms of the disease and point out altered expression of mitochondrial transport proteins in human AD brains, mostly contributed by APP-CTFs.

## Discussion

In the current study, we unraveled several alterations in the expression and localization of different components of the mitochondrial transport machinery in cellular and mice AD models and in human post-mortem SAD brains. We reported that depletion of the endogenous full-length APP is associated with a reduced mitochondria movement that is most likely linked to an enhanced expression of the stop protein SNPH and to reduced expression of dynein (lC 1,2) motor. Moreover, the enhanced expression of Miro1, TRAK1, TRAK2, and Kif5 (A, B, C) in APPKO cells further highlights a potential direct or indirect role for the endogenous APP in the control of mitochondrial transport proteins. In fact, previous studies have shown that vesicular APP is transported along axons and that APP directly interacts with Disrupted-In-SChizophrenia 1 (DISC1), shown to regulate anterograde mitochondrial transport and to interact with Miro1 and TRAK1/2 [40]. In addition, APP also interacts with the kinesin light chain subunit KLC1 (Kinesin light chain 1) and acts as a conventional cargo for kinesin-I microtubule motor protein [41].

We carried out experiments to investigate the specific impact of the accumulation of APP-derived fragments on mitochondrial transport components and used a cellular model overexpressing an AD familial mutation (SH-SY5Y APPswe). This cellular model combined with pharmacological modulation of β- and γ-secretase activities allows us to demonstrate that both Aβ and C99 contribute to the alteration of the expression of mitochondrial transport components. As a matter of fact, the reduction in the expression of Miro1 and TRAK1 in APPswe cells is maintained upon γ-secretase inhibition that blocks Aβ production and exacerbates APP-CTFs overaccumulation. Moreover, we reported a reduction in the expression of SNPH and of full-length Kif5 (A, B, C) in APPswe cells treated with the γ-secretase inhibitor. Our results in AAV-C99 mice brains further support a pathogenic molecular signature of the mitochondrial transport proteins linked to C99 accumulation in vivo.

The defect of mitochondrial movement and the alterations of the expression of transport proteins in PSDKO cells could likely be associated to APP-CTFs accumulation. However, we must not exclude that the defects reported in this model could occur in a γ-secretase-independent manner, through PS-mediated control of vesicular transport and trafficking [42]. This assumption is supported by a pioneer study by Kamal et al. demonstrating that vesicular PS1 is transported with APP along axons by kinesin 1 [41]. In light of our findings and this observation, one may speculate that PS depletion (or loss of function linked to familial forms of AD) could destabilize vesicular transport and impact global transport machinery.

Our experiments in APPswe cells revealed an increase in the expression of Kif5 (low band) and IC 1,2 that seems to be linked with Aβ production since these alterations are reversed by γ-secretase inhibition. Consistent with a specific toxic effect of Aβ on transport machinery, a recent study reported a reduction of Miro1 level by Aβo [43]. Accordingly, we reported a reduction of Miro1 colocalization with mitochondria in differentiated neuroblastoma cells treated with Aβo.

Previous studies have suggested that APP axonal transport by kinesin-I may interfere with vesicular APP processing to Aβ and APP-CTFs occurring in an axonal membrane compartment containing β-secretase and PS1 [41]. A second study demonstrated that knock-down of DISC1, is accompanied by an increase in APP-CTFα and a decrease in Aβ42 and Aβ40 levels [44]. Our findings provide original observations demonstrating that endogenous full-length APP as well as the accumulation of APP-derived fragments occurring under AD conditions interfere in turn with transport machinery. Since these alterations affect both dynein and kinesin motors and implicate several mitochondrial adapters as well as the stop protein SNPH, we may hypothesize that APP and its derived fragments impact global anterograde and retrograde mitochondrial transport under physiological and AD conditions.

Although differentiated neuroblastoma cells are not fully functional neurons, they are an alternative study system mimicking polarized primary culture of neurons. Moreover, differentiated SH-SY5Y APPswe cells still overproduce APP-derived fragments, the accumulation of which is enhanced by γ-secretase inhibitor. Using these models, we demonstrated that the loss of the localization of the transport proteins to mitochondria is an additional molecular event regulated by APP-derived fragments (C99, C83, and Aβ) and likely perturbing mitochondria anterograde and retrograde transport. Importantly, we also demonstrate the impact of APP-CTFs accumulation on the localization of mitochondria and transport machinery in murine primary neuron cultures. These data fully consolidate our biochemical data demonstrating an alteration of the expression of a set of mitochondrial transport machinery components linked to APPswe overexpression and concomitantly implicating C99 and Aβ accumulation.

Mitophagy failure is commonly associated with several AD-related proteins (Tau, Aβ, APP-CTFs, and APOE) [45] and is likely associated with a defect of mitochondrial transport along axons. Importantly, genetic deletion of Miro1 or the blockade of Miro1 ubiquitination and subsequent degradation leads to a delayed translocation of the E3 ubiquitin ligase Parkin onto damaged mitochondria and reduced mitochondrial clearance by mitophagy in both fibroblasts and cultured neurons [46]. On the other side, Pink1 and Parkin act in concert to target Miro1 to proteasomal degradation to arrest mitochondria motility [47, 48]. These observations and other studies have proposed Miro1 as a molecular marker for the risk of Parkinson’s disease and as a potential therapeutic target for the disease [49, 50]. Interestingly, our data revealed that Miro1 expression is drastically reduced in APPswe cells and is also modulated in AAV-C99 and 3xTgAD-aged mice brains. We also revealed that Miro1 localization to mitochondria is impacted by APP-CTFs accumulation and Aβo treatment in differentiated neuroblastoma cells. These observations place Miro1 as a key molecular actor likely controlling both mitochondrial transport and mitophagy defects occurring in AD.

We reported that SNPH expression is reduced in concert with APP-CTFs accumulation in APPswe cells, in aged 3xTgAD mice (13-month-old) hippocampi and AAV-C99 injected mice brains and also noticed a slight decrease in human SAD brains at advanced stages of the disease. Moreover, the reduced expression level of SNPH in human brains significantly correlates with APP-CTFs and to a lesser extent with Aβ and pathogenic pTau (T231 and S422). SNPH deficit may likely contribute to AD development or progression. In fact, SNPH is linked to synaptic dysfunction accompanying the loss of static mitochondria [4]. On the contrary, enhanced levels of SNPH in young 3xTgAD mice could be taken as a compensatory mechanism that ensures local mitochondria movement arrest allowing calcium buffering and ATP release at the level of altered synapses [51]. In fact, previous studies reported that SNPH expression increases in mature neurons in mice [52] and plays an essential role in synaptic plasticity and in the stimulation of the liberation of synaptic vesicles [53, 54].

TRAK1/2 adapters coordinate opposing motors (i.e., kinesin-1 and dynein) for the proper transport of mitochondria [6]. While a previous study has shown that TRAK2 protein level is reduced by caspase3-truncated Tau [55], the specific impact of Aβ and APP-CTFs was unknown. We unraveled herein a reduction of the expression and the localization in mitochondria of TRAK1 and/or TRAK2 linked to APP-CTFs accumulation and/or Aβo treatment. Importantly, our results in human brains at late AD stages support a reduction of TRAK1/2 expression linked to AD progression that correlates with Aβ (TRAK2) or APP-CTFs (TRAK1).

The contribution of the kinesin Kif5 (A, B, C) to mitochondria transport defect in AD is still not clear. While a previous study showed enhanced Kif5A levels at late Braak stages [56], a second paper reported a diminution of Kif5A by Aβ [57]. Our results show on the one hand an Aβ-dependent Kif5 (A, B, C) down-expression in APPswe cells and on the other hand that the lesser localization of KiF5 (A, B, C) to mitochondria is linked to both APP-CTFs and Aβ. Strikingly, we did not observe any significant alteration of the expression of Kif5 (A, B, C) or its cleaved form in human post-mortem brains cohort. Moreover, while IC1, 2 down expression positively correlates with TRAK2 and Kif5 (A, B, C) levels, it did not correlate with any of AD-related proteins. These observations may question the genuine role of Kif5 (A, B, C) and IC1,2 in AD development.

We and other laboratories have reported that 3xTgAD mice models develop in an age-dependent manner alteration of synaptic markers, synaptic plasticity, and cognitive deficits [58, 59]. Similarly, we previously reported synaptic plasticity deficit (i.e., reduced long-term potentiation) in AAV-C99 mice [22] and demonstrated a link between APP-CTFs accumulation and decreased spontaneous activity, altered LTP and learning deficits in 3xTgAD mice [18]. Moreover, our recent study reported altered mitochondria structure in neurons and of autophagy/mitophagy in brains of 3xTgAD (at 5-6 months old) and AAV-C99 mice (at both 2–3 and 12 months old) [23]. Decreased mitochondria respiration was also described in young 3xTgAD mice at 3 months old [60]. These results strongly suggest that altered neuronal function and cognitive deficits in our mice models are likely linked to altered mitochondria function and to the accumulation of dysfunctional mitochondria. The current study reports a dysregulation of the expression of key mitochondrial transport proteins (in cells, two different AD murine models, and in human SAD brains) and an altered colocalization of mitochondria with transport machinery (in differentiated neuroblastoma model and murine primary neurons). These results led us to assume that altered mitochondria transport likely contributes to the accumulation of dysfunctional mitochondria (defect of retrograde transport) and to neuronal demise (anterograde transport) in our AD murine models. However, we still need a dedicated study to fully demonstrate the functional link between mitochondrial transport and neuronal plasticity.

Together, our data unravel novel molecular mechanisms impacting mitochondria transport, the defect of which may contribute to the loss of the transport of dysfunctional mitochondria to their sites for degradation by mitophagy and of healthy ones for the delivery of ATP to synapses. This study will certainly stimulate further research targeting mitochondria transport machinery as a means to modulate simultaneously defects of mitochondria movement and mitophagy processes occurring in AD.

### Supplementary information

Supplementary Information

Original Western blots

Supplementary video 1

Supplementary vide2

Supplementary vide3

Supplementary vide4
