# Supplementary material for: The amyloid precursor protein and its derived fragments concomitantly contribute to the alterations of mitochondrial transport machinery in Alzheimer’s disease
Source: Cell Death Dis. 2024 May 28;15(5):367. doi: 10.1038/s41419-024-06742-2 (PMC11133367; doi:10.1038/s41419-024-06742-2)

Supplementary Information

Supplementary methods

Mitochondrial movement study

MEF were transfected at 50-60% confluence with the mitochondrial RFP probe (mitRFP) (34) using lipofectamine 2000 (Invitrogen 11668-019). The medium was replaced 6 hours post-transfection and cells were observed 36h later with the LSM 780 microscope. Cells were mounted in a chamber using Krebs Ring Buffer (135mM NaCl, 5mM KCl, 1mM $MgSO_4$, 0.4mM $K2HPO_4$, and 20mM HEPES, pH 7.4) complemented with 1mg/ml D-Glucose and 1µM Ca^{2+}. We captured time laps images of mitochondria (18 cycles of 1 second at a resolution of 1024 x 1024 pixels). Analysis were then made with a macro on ImageJ as already described (34).

Aβ production

Dry Aβ 1-42 (Bachem AG 4014447.1000) (1mg) was diluted in HFIP (1, 1, 1, 3, 3, 3, 3 – Hexafluoro-2-propanol) (SIGMA 105228-5G) for 1 hour to obtain a 1mM homogenous solution, then aliquoted into microfuge tubes and lyophilized under laminar hood overnight (O/N) to ensure complete evaporation. To form Aβ oligomers, peptide films were resuspended to 5mM in DMSO, diluted in ice cold PBS. Aggregation was allowed to proceed for 24 hours at 4°C before the peptide solution was centrifuged at 14000 x g for 10 minutes at 4°C. Supernatant containing Oligomeric Aβ (Aβo) was used at ≈ 5µM for 16 hours.

Tau species quantification ELISA

For pT231-Tau human ELISA, steps were performed according to the manufacturer's instructions (Invitrogen). Samples were diluted at 1:50 in sample diluent of the kit. For total pS422-Tau ELISA, we developed a homemade test with the anti-pSer422 monoclonal antibody (clone 2H9, 4BDX-1501, SPQI-4BioDx, France) as the capture antibody and an anti-amino-terminal home-made antibody (against amino-acids23-30) as the phase antibody. Following incubation with an anti-IgG2b HRP conjugated antibody (Southern Biotech) for1 hour and Tetramethyl benzidine (Sigma-Aldrich) revelation, plates were measured, after sulfuric acid addition, with a spectrophotometer (Multiskan FC, Thermo) at 450 nm.

Legend to supplementary figures

Supplementary video 1. Time laps showing mitochondria movement in MEF APPWT and MEF APPKO

Supplementary video 2. Time laps showing mitochondria movement in MEF PSWT and MEF PSDKO

Supplementary Fig. 1. The graphs show mitochondrial movement expressed as mitochondrial motile fraction. **A** in MEF APPWT and MEF APPKO. **B** in MEF PSWT and MEF PSDKO. ** $P < 0.01$ versus MEF APPWT using Mann-Whitney's test. **** $P < 0.0001$ versus MEF PSWT using Mann-Whitney's test.

Supplementary Fig. 2. β-secretase inhibition in APPswe cells do not impact the expression of mitochondrial transport proteins. **A** SDS-PAGE showing the accumulation of C99 and C83 in APPswe SH-SY5Y cells upon γ-secretase inhibition (γ-sec inh) and the specific accumulation of C83 and the reduction of C99 levels in APPswe SH-SY5Y cells upon β-secretase inhibition (β-sec inh). Full-length APP is shown as loading control. **B** SDS-PAGE of SNPH, Miro1, TRAK1, TRAK2, Kif5 (A, B, C), Kif5 (low), and IC 1,2 in APPswe treated with vehicle or with β-secretase inhibitor. Representative SDS PAGE of β-actin are shown as loading controls. Full-length western blots are also provided in a separate supplementary file.

Supplementary Fig. 3. SH-SY5Y expressing empty vector (control) treated with the γ-secretase inhibitor do not show any modulations of the expression of the mitochondrial transport proteins. **A-C** Quantitative graphs of SNPH (b), Miro1, TRAK1, and TRAK2 (c), and Kif5 (A, B, C), Kif5 (low) and IC1, 2 (d) protein levels expressed as means ± SEM of control + Veh (set at 100%). Data were obtained in 5-6 independent experiments. The representative SDS-PAGE of SNPH, Miro1, TRAK1 and TRAK2, Kif5 (A, B, C), Kif5 (low), and IC1, 2 in control cells treated with vehicle (Veh) or with γ-secretase inhibitor (γ-sec inh) is shown in Fig. 3. Full-length western blots are also provided in a separate supplementary file. ns: not significant.

Supplementary Fig. 4. MEFs APPKO treated with γ-secretase inhibitor do not show any modulations of the expression of mitochondrial transport proteins. **A** SDS-PAGE of SNPH, Miro1, TRAK1 and TRAK2, Kif5 (A, B, C), IC 1, 2 in MEFs APPWT and MEFs APPKO. β-actin were revealed as loading control. MEF APPKO treated with vehicle or with γ-sec inh were loaded in the same gels. Dashed line indicates that unrelated samples were loaded between the two conditions and are not shown. Full-length western blots are also provided in

a separate supplementary file. **B-D** Quantitative graphs of SNPH (B), Miro1, TRAK1, and TRAK2 (C), and Kif5 (A, B, C), Kif5 (low) and IC1, 2 (D) protein levels expressed as means ± SEM of MEFs APPWT (set at 100%). Data were obtained from 4 independent experiments. ns: not significant.

Supplementary Fig. 5. Representative Immunofluorescences images of differentiated SH-SY5Y control and APPswe cells stained at day 20 post-differentiation with β3-tubulin, MAP2, NeuN, and Tyrosine Hydroxylase (shown in green). Nuclei are stained with Dapi. Scale bar = 30µm.

Supplementary Fig. 6. β -secretase inhibition alters the localization of mitochondrial transport proteins with mitochondria. **A** Representative immunofluorescence images showing the colocalization of transport proteins (Miro1, TRAK1, TRAK2 and IC1,2 in red) with mitochondria (TOMM20 in green) in differentiated APPswe SH-SY5Y cells treated with vehicle (Veh) or with with β-secretase inhibitor (β-sec inh). Scale bar = 5µm. **B-D** Quantitative graphs of the Pearson's coefficient representing the colocalization (arrows) of SNPH and Miro1 (B), TRAK1 and TRAK2 (C), and Kif5 and IC1, 2 (D) with mitochondria. Graphs are expressed as means ± SEM. Data were obtained from 4 independent experiments. * $P < 0.05$, *** $P < 0.001$, and **** $P < 0.0001$ and ns: not significant versus APPswe cells treated with vehicle using Mann-Whitney's test.

Supplementary Fig. 7. Mitochondrial transport proteins colocalization with mitochondria is affected by Aβ oligomers (Aβo). **A** Representative immunofluorescence images showing the colocalization of transport proteins (SNPH, Miro1, and TRAk1 in red) with mitochondria (TOMM20 in green) in differentiated SH-SY5Y control cells non-treated (Control) or treated with Aβo**.** Scale bar = 5µm. **B-D** Quantitative graphs of the Pearson's coefficient representing the colocalization (arrows) of SNPH and Miro1 (B), TRAK1 and TRAK2 (C), and Kif5 and IC1, 2 (D) with mitochondria. Graphs are expressed as means ± SEM. Data were obtained from 4 independent experiments. * $P < 0.05$, ** $P < 0.01$, and ns: non-significant versus control using Mann-Whitney's test.

Supplementary Fig. 8. A Representative SDS-PAGE showing the expression of APP, and APP-CTFs in human AD brains. A representative SDS PAGE of β-actin is shown as loading controls. Full-length western blots are provided in a separate supplementary file. **B** Expression levels of pTauT231 and pTauS422 analyzed by ELISA on total brain extracts (control n= 4 and AD IV-VI, n=12). quantitative graph indicates means pTau forms

levels versus total Tau ± SEM versus controls (taken as 100%). **C** Heat map of the correlation matrix computing Spearman r value for every pair of data sets is displayed in each box. Color scale of Spearman r values where 1 represents the maximum positive correlation value and -1 represents the maximum negative correlation value, and 0 represents no correlation. B * $P < 0.05$, and ns: not significant versus control using Kruskal-Wallis test.

Supplementary table 1: List of antibodies and corresponding dilutions used in the study.

**Antibodies**	**Dilution**		**Host**	**Reference**	**Supplier**
	**WB**	**IF**			
β-Actin	1/5000	/	Mouse	A5316	Sigma
SNPH	1/1000	1/200	Rabbit	PA5-20529	ThermoFisher scientific
Miro1/RhoT1	1/1000	1/200	Rabbit	PA5-42646	Invitrogen
TRAK1	1/1000	1/200	Rabbit	HPA005853	Merck
TRAK2	1/1000	1/200	Rabbit	HPA015827	Merck
IC1,2	1/1000	1/200	Mouse	sc-13524	Santa Cruz Biotechnology
Kif5 A, B, C	1/1000	1/200	Rabbit	ab62104	Abcam
APP-Cter	1/1000	1/1000	Rabbit	Gift from Dr. Paul Fraser, Toronto, Canada	
TOMM20	/	1/500	Mouse	612278	BD transduction
CoxIV	/	1/400	Rabbit	mAb 3E11 #4850	Cell signaling
Tyrosine Hydroxylase	/	1/1000	Rabbit	GTX113016	GeneTex
NeuN	/	1/500	Rabbit	ab177487	Abcam
β3-Tubulin	/	1/2000	Mouse	MMS-435P	Covance
MAP 2	/	1/1000	Guinea Pig	Cat# 188,004, RRID:AB_2138181	Synaptic Systems
PS1	1/1000	/	Rabbit	D39D1	Cell Signaling
PS2	1/1000	/	Rabbit	D30G3	Cell Signaling

Vaillant-Beuchot L. et al. Supplementary Fig. 1

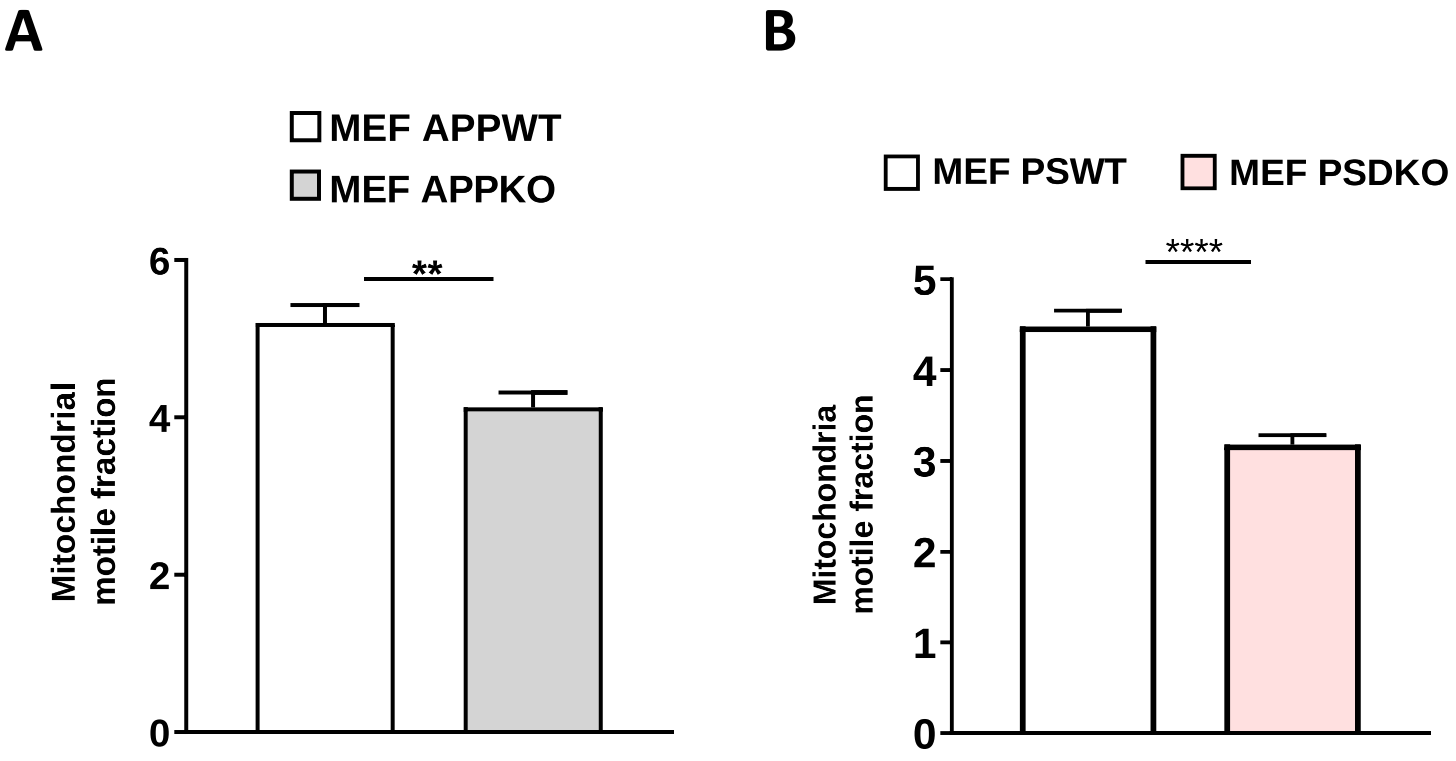

Vaillant-Beuchot L. et al. Supplementary Fig. 2

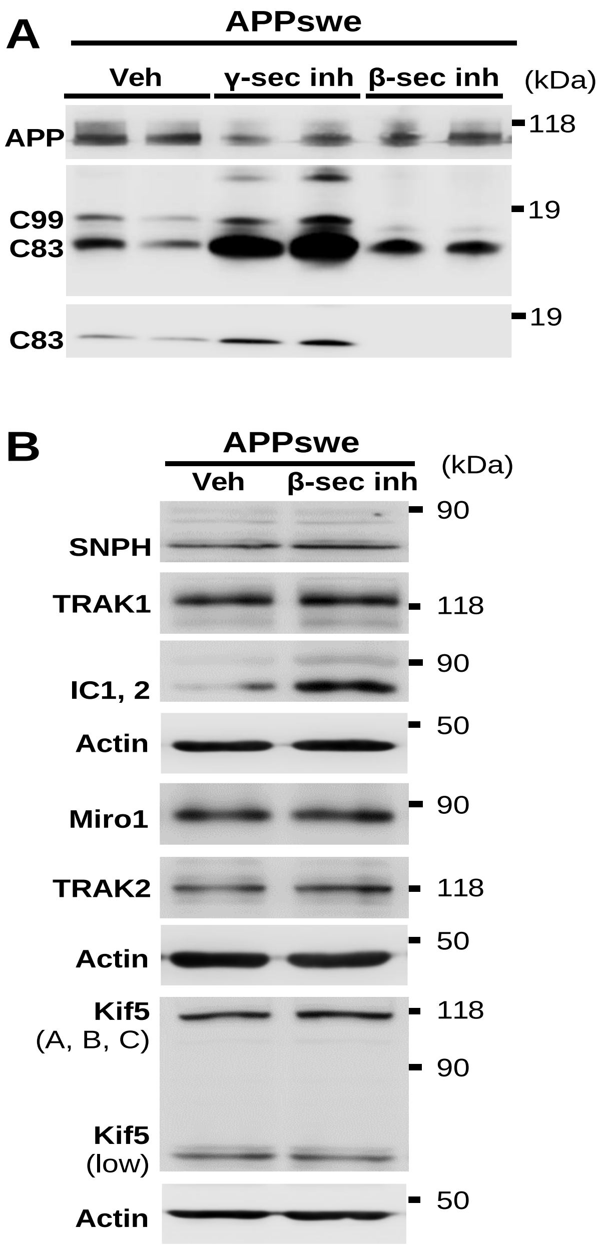

Vaillant-Beuchot L. et al. Supplementary Fig. 3

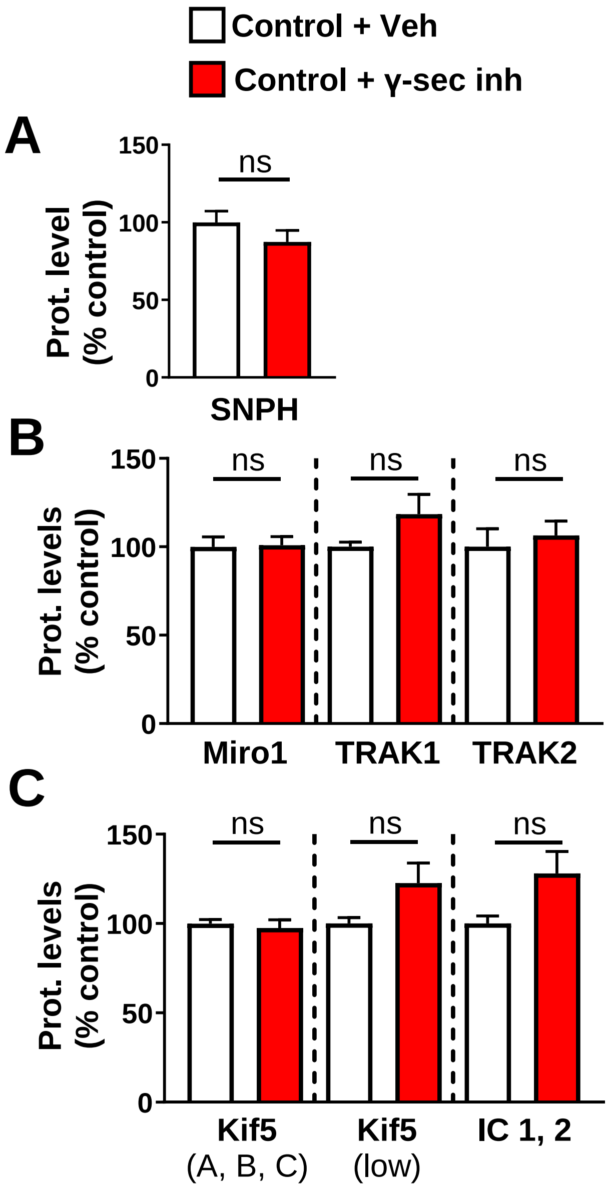

Vaillant-Beuchot L. et al. Supplementary Fig. 4

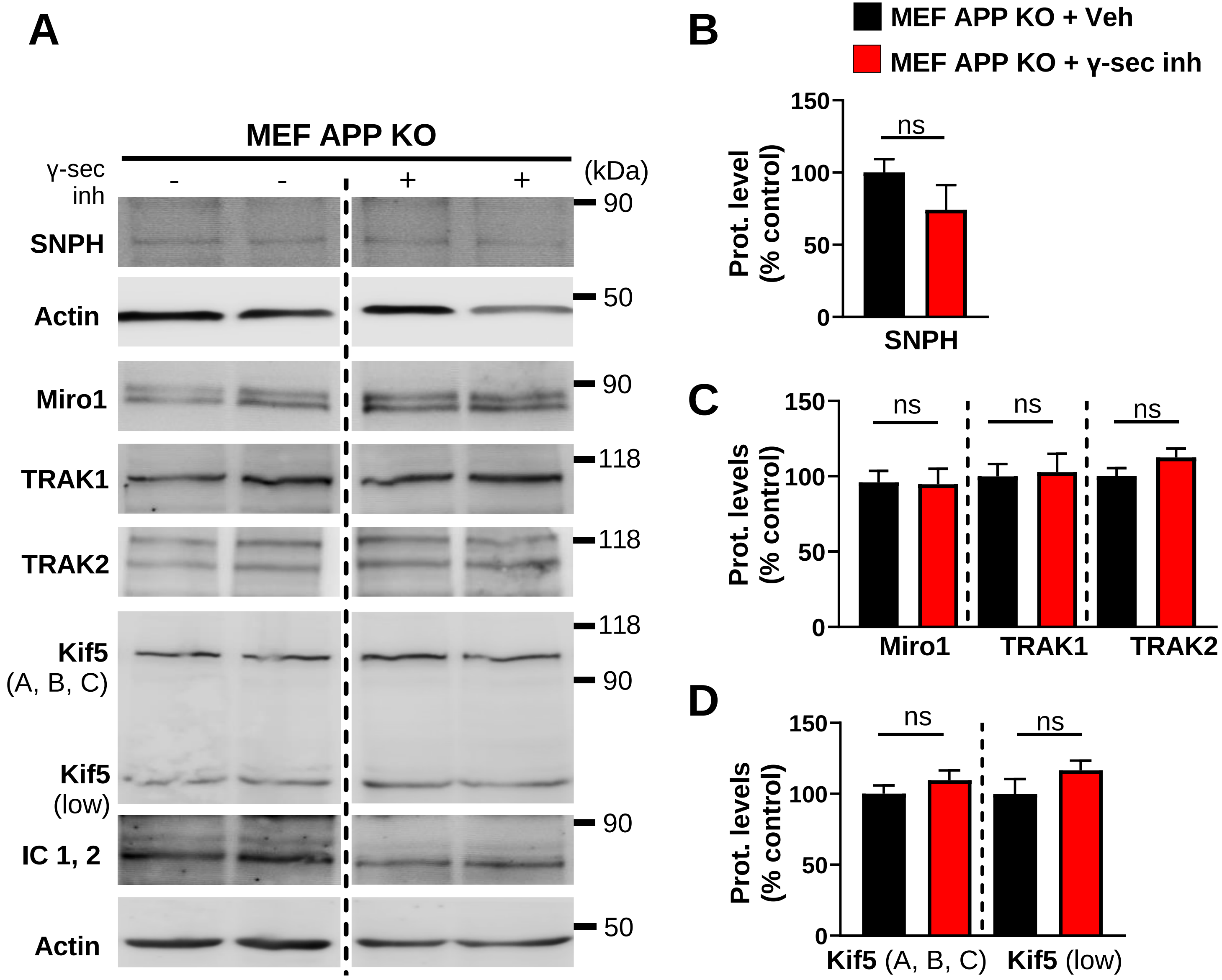

Vaillant-Beuchot L. et al. Supplementary Fig. 5

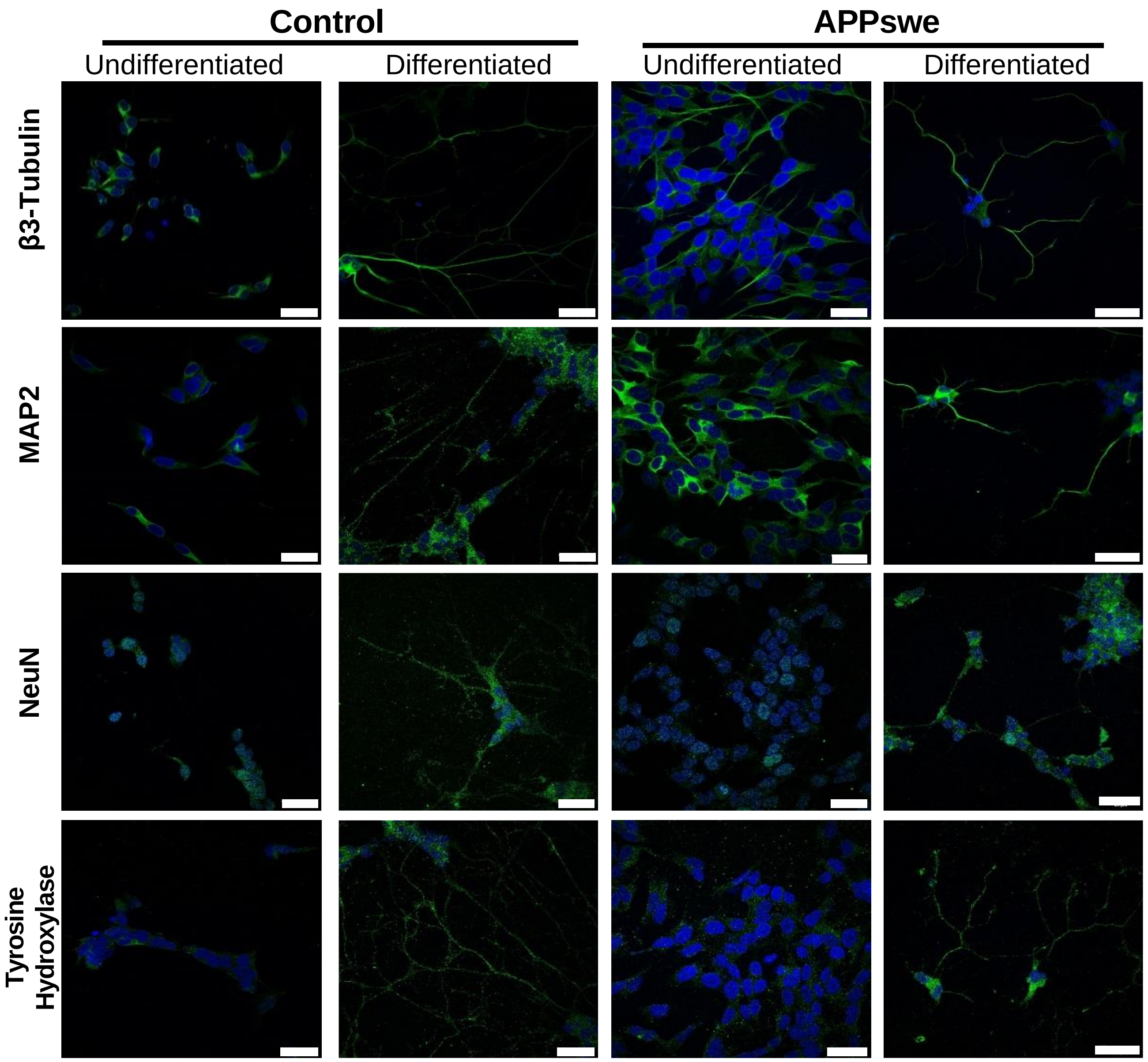

Vaillant-Beuchot L. et al. Supplementary Fig. 6

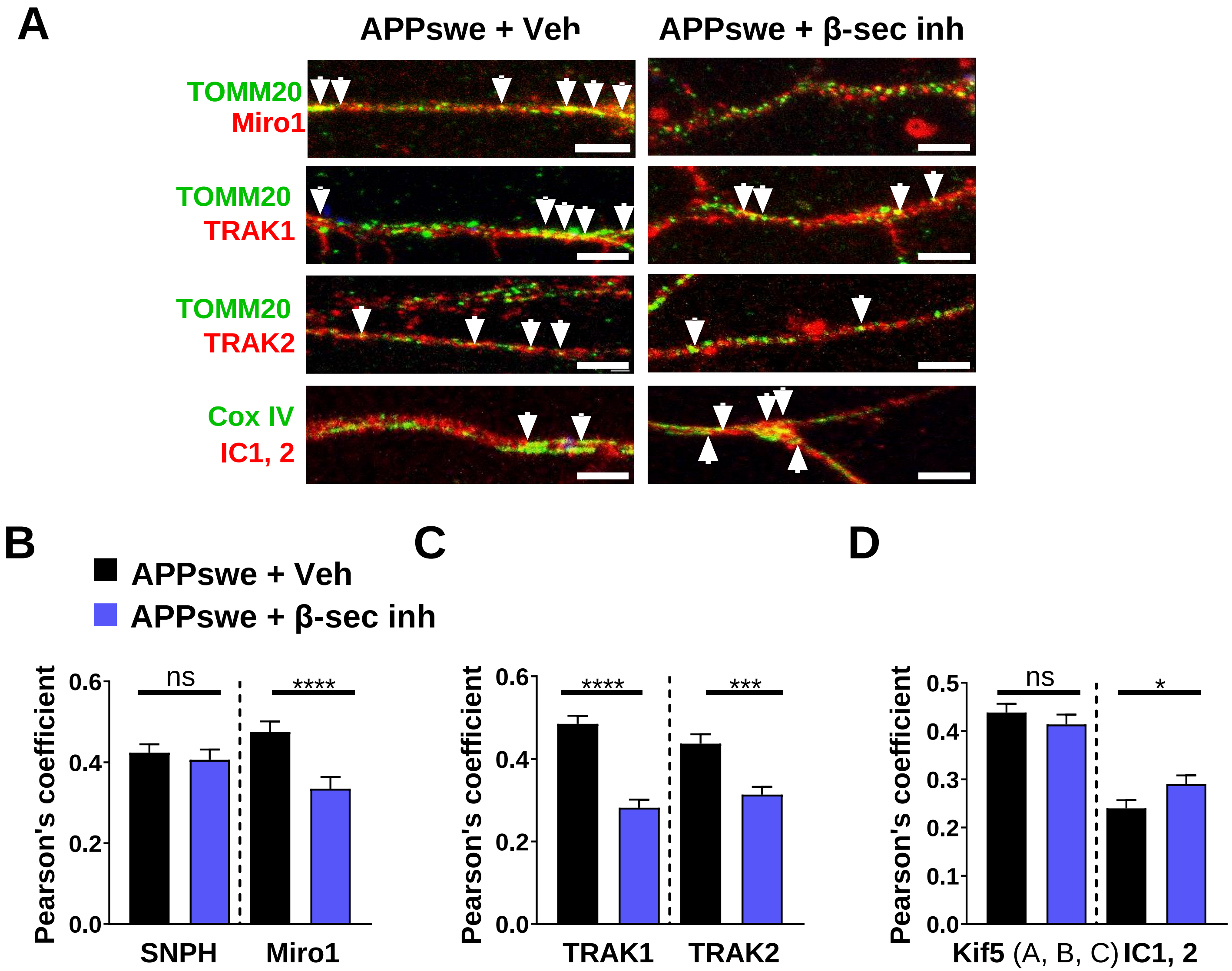

Vaillant-Beuchot L. et al. Supplementary Fig. 7

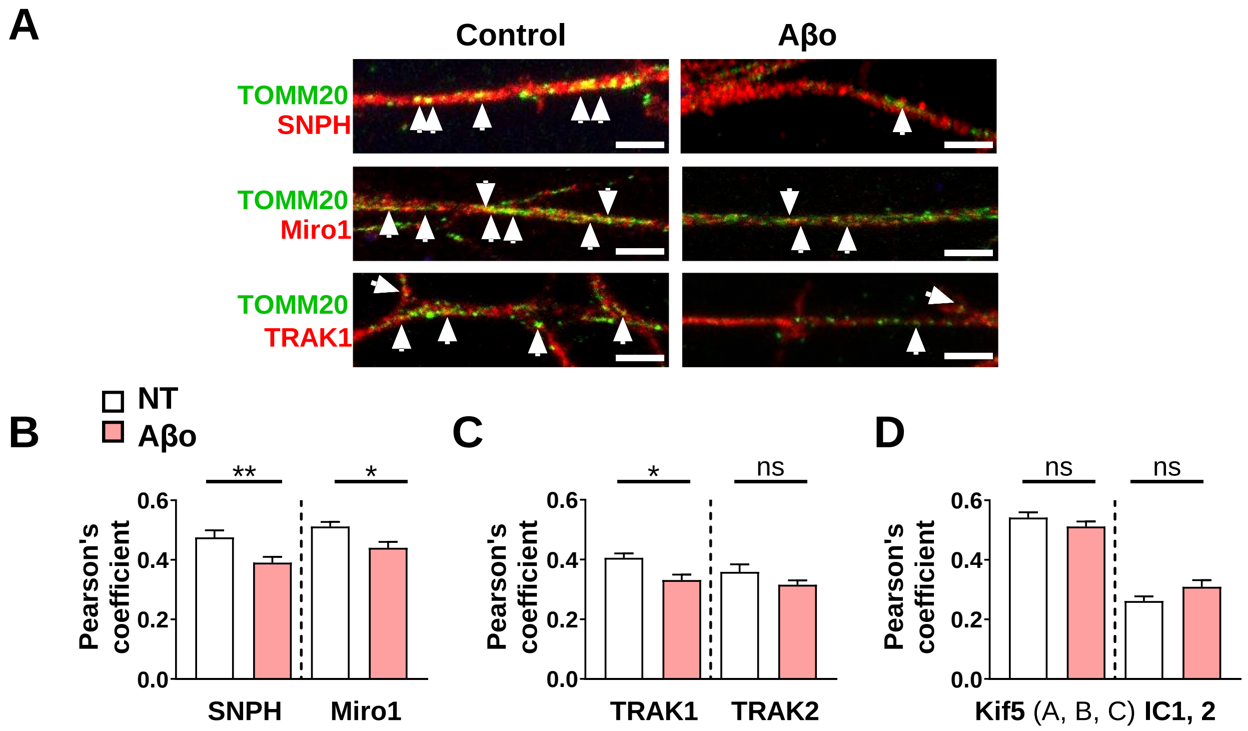

Vaillant-Beuchot L. et al. Supplementary Fig. 8

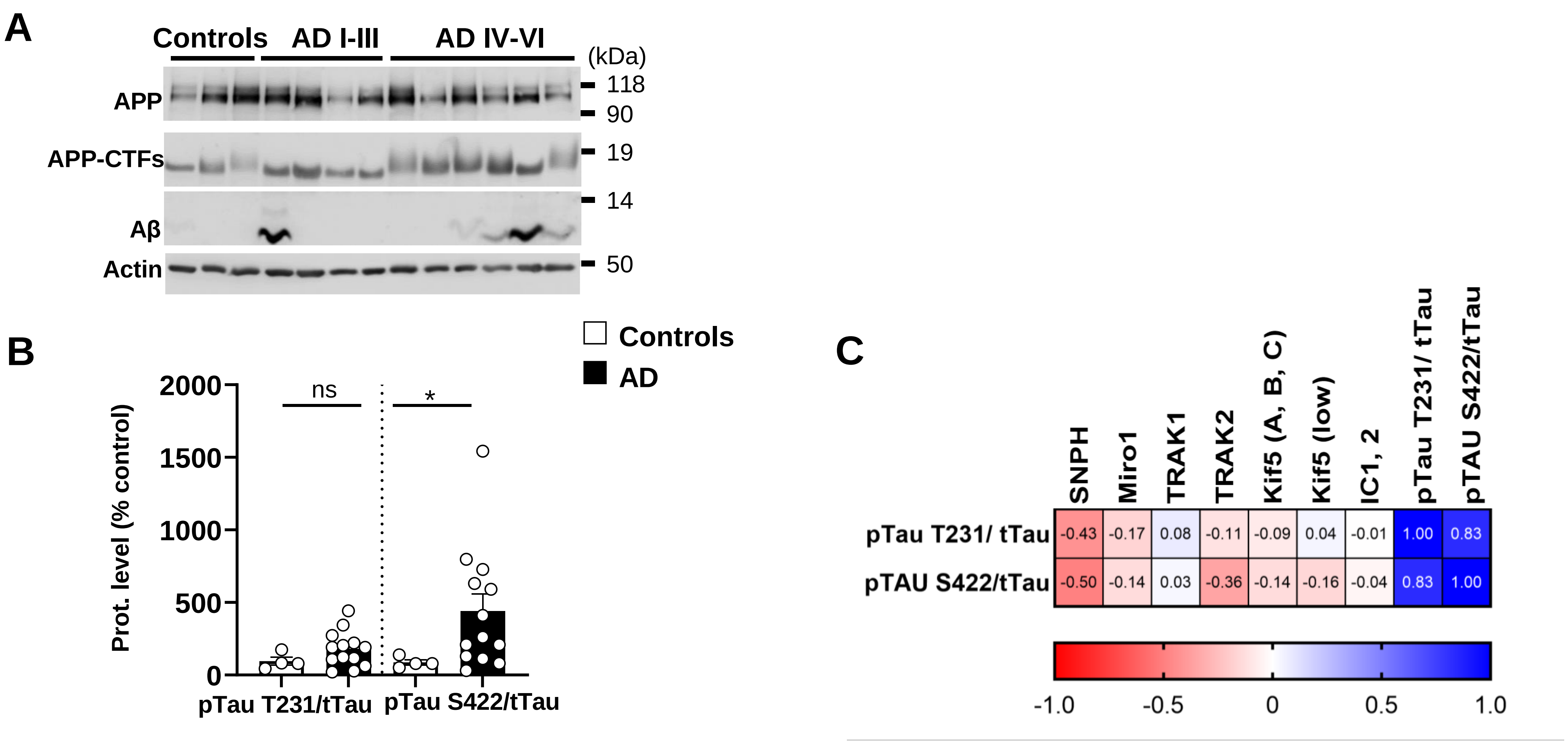

Supplement: Supplementary file 1 — Supplementary Information [file 41419_2024_6742_MOESM1_ESM.pdf]
