# Supplementary material for: The amyloid precursor protein and its derived fragments concomitantly contribute to the alterations of mitochondrial transport machinery in Alzheimer’s disease
Source: Cell Death Dis. 2024 May 28;15(5):367. doi: 10.1038/s41419-024-06742-2 (PMC11133367; doi:10.1038/s41419-024-06742-2)
Supplement: Supplementary file 2 — Original Western blots [file 41419_2024_6742_MOESM2_ESM.pdf]

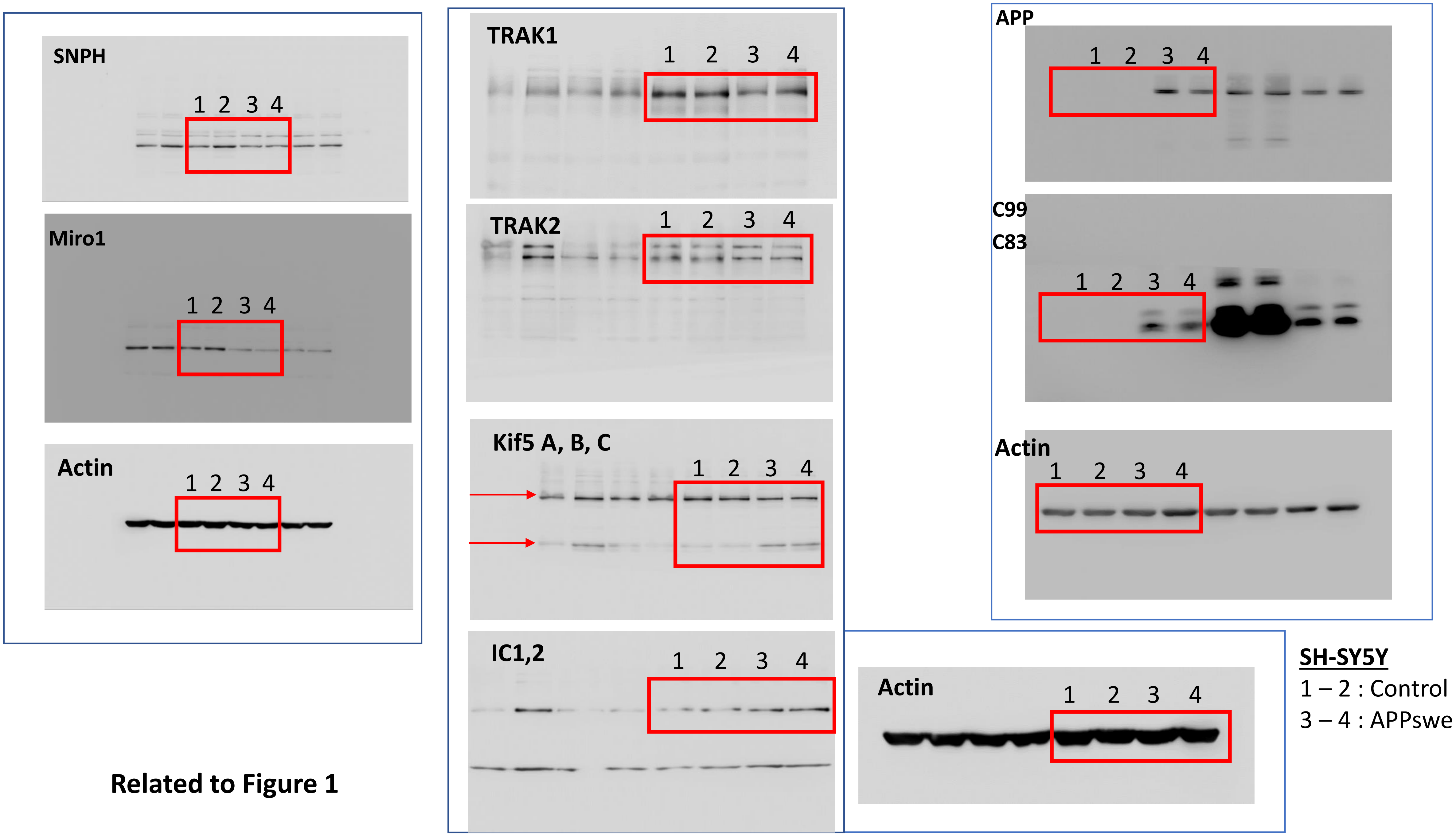

Related to Figure 1

Related to Figure 2

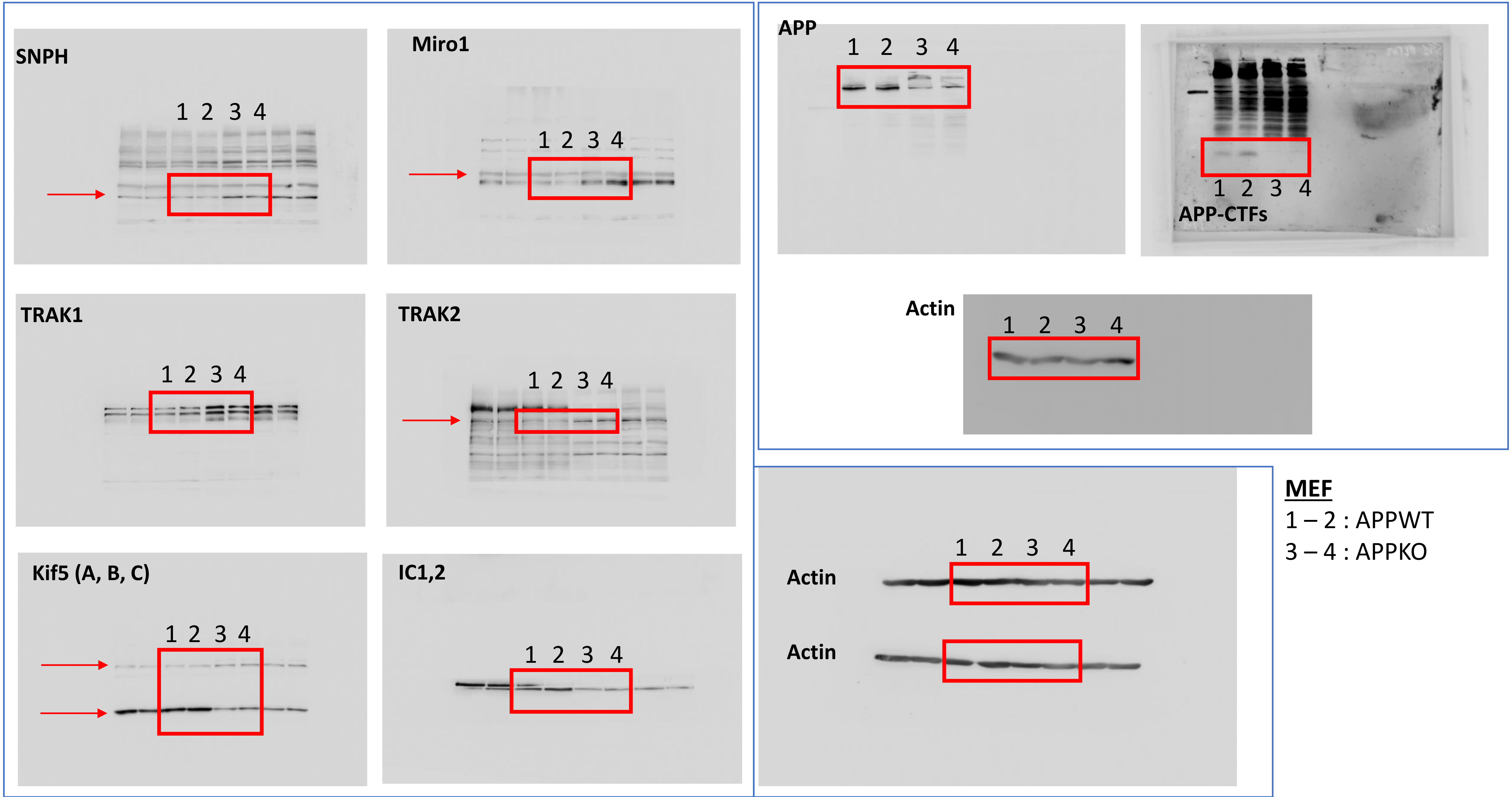

Related to Figure 3

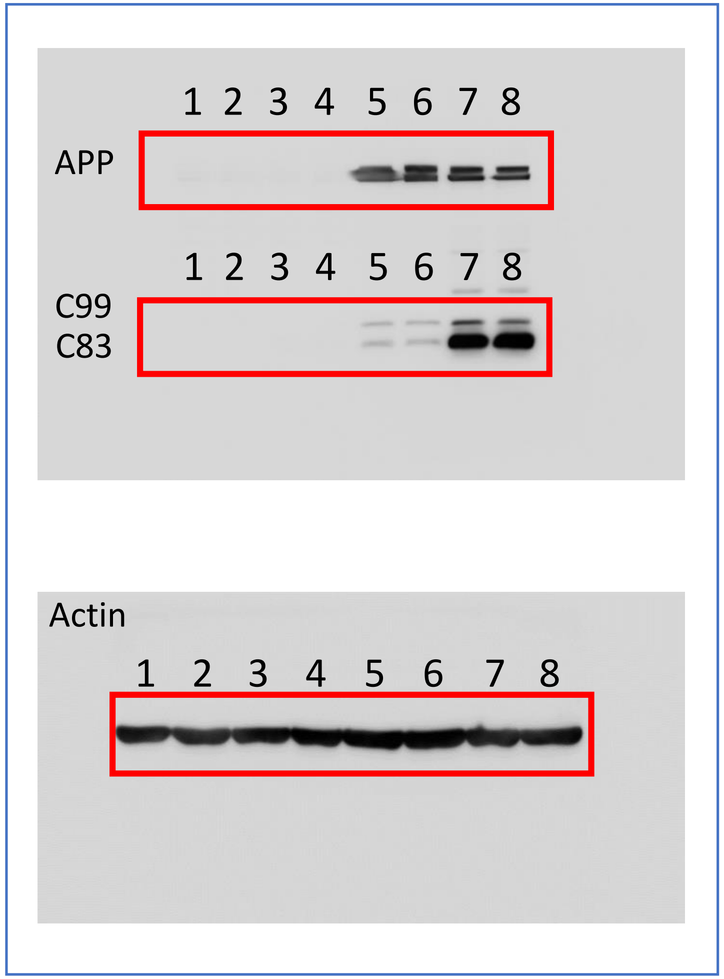

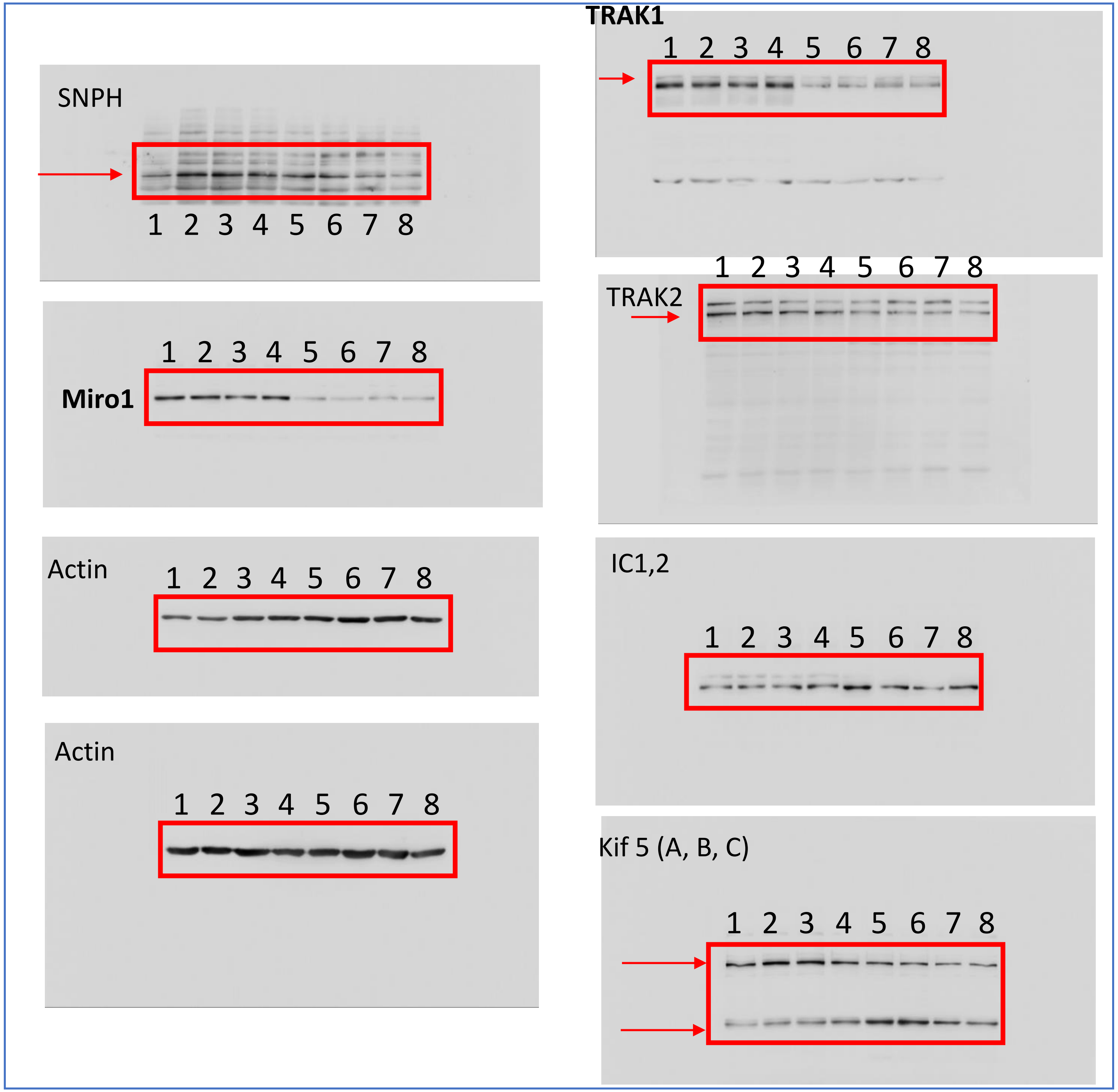

SH-SY5Y

1 – 2 : Control + Veh

3 – 4 : Control + γ-secretase inhibitor

5 – 6 : APPswe + Veh

7 – 8 : APPswe + γ-secretase inhibitor

Related to Figure 4

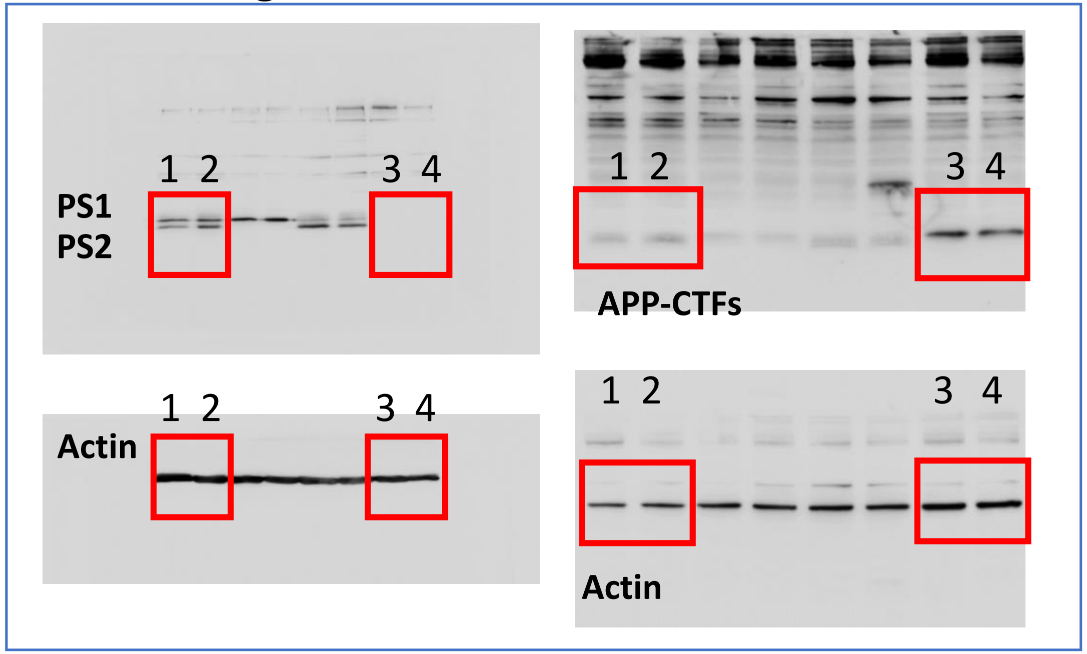

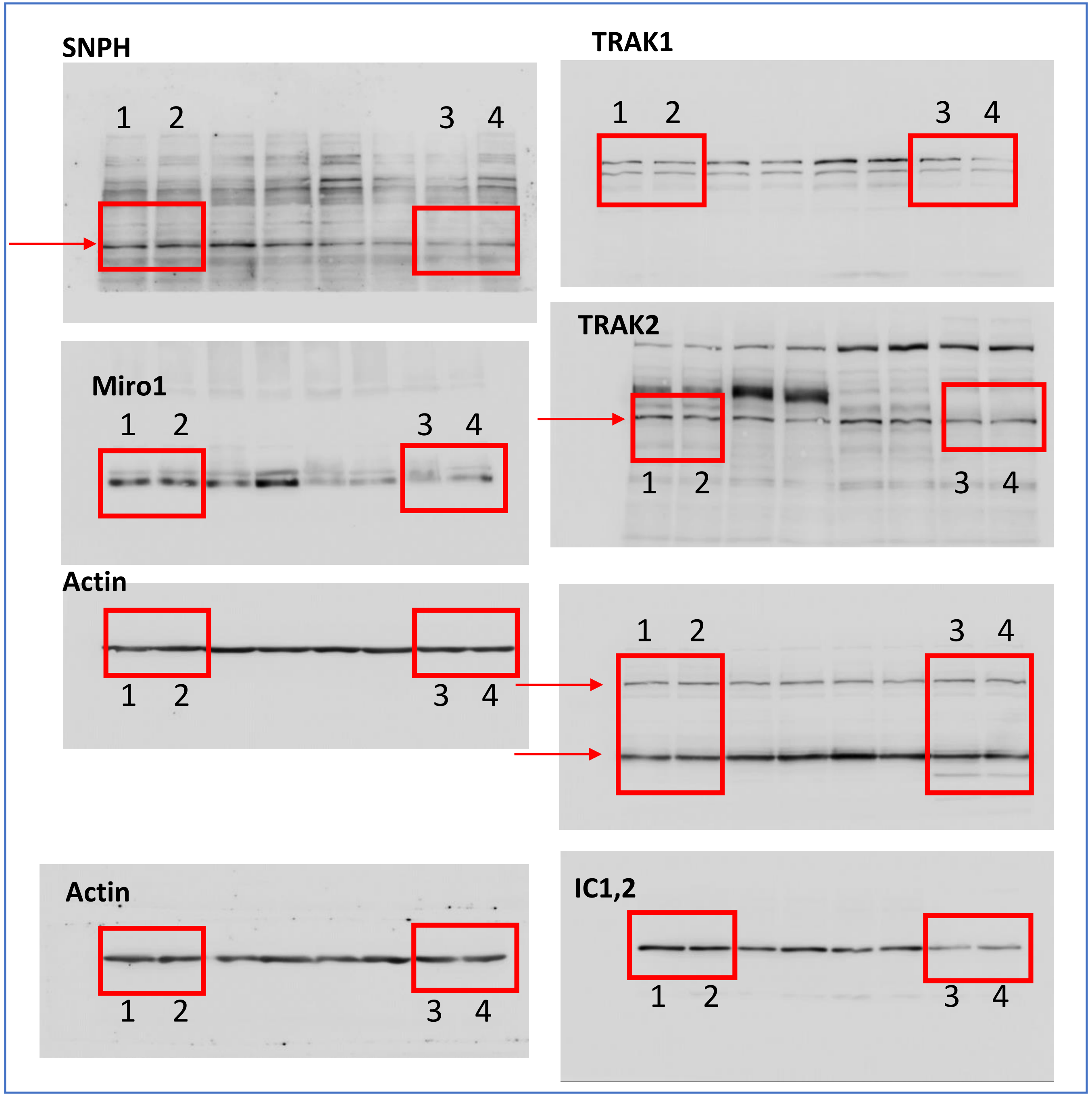

MEF

1 – 2 : PSWT

3 – 4 : PSKO

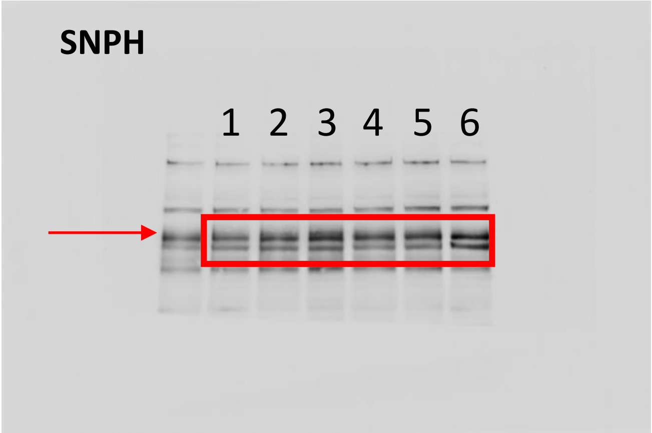

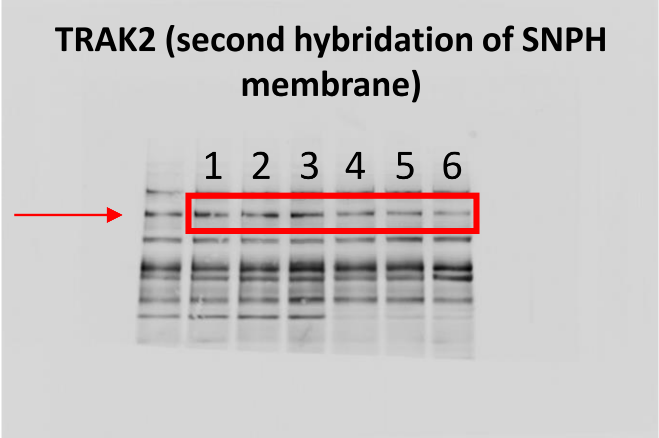

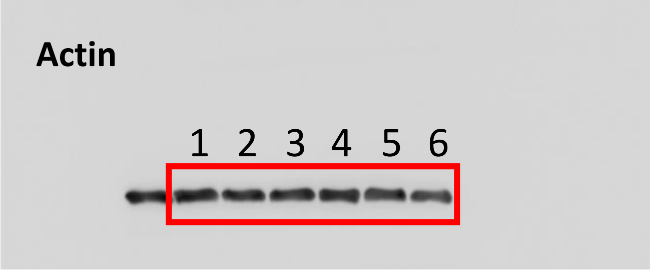

<u>4 months mice</u>

1, 2, 3 : WT

4, 5, 6 : 3xTgAD

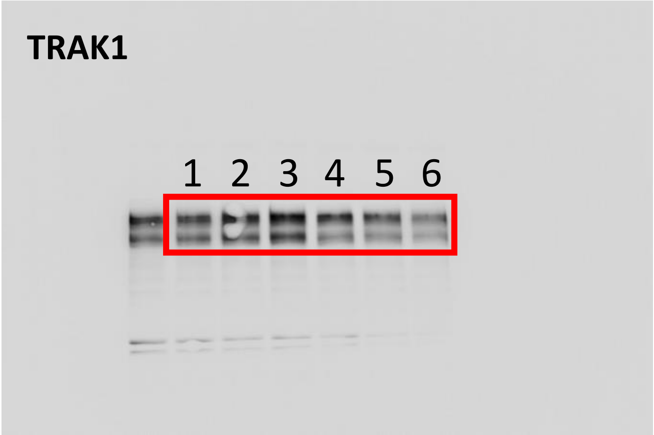

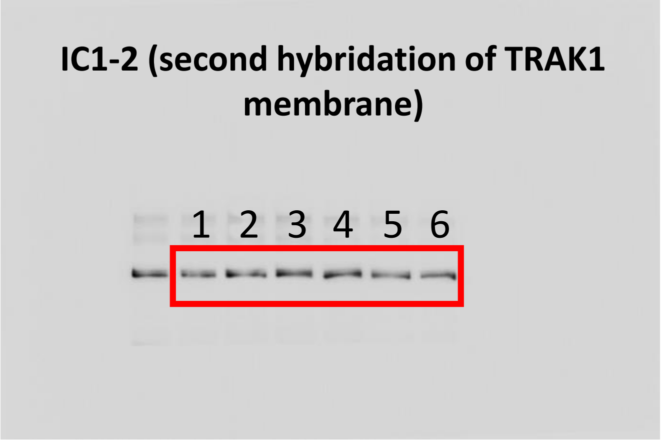

Related to Figure 7a

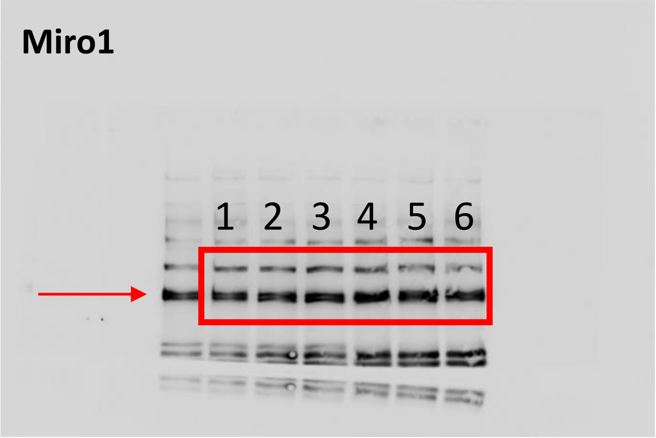

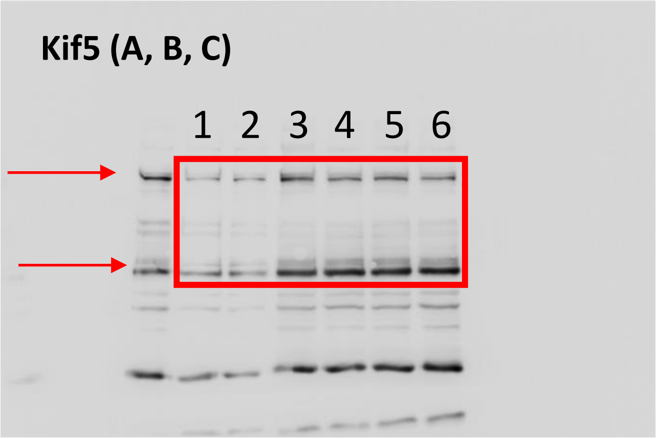

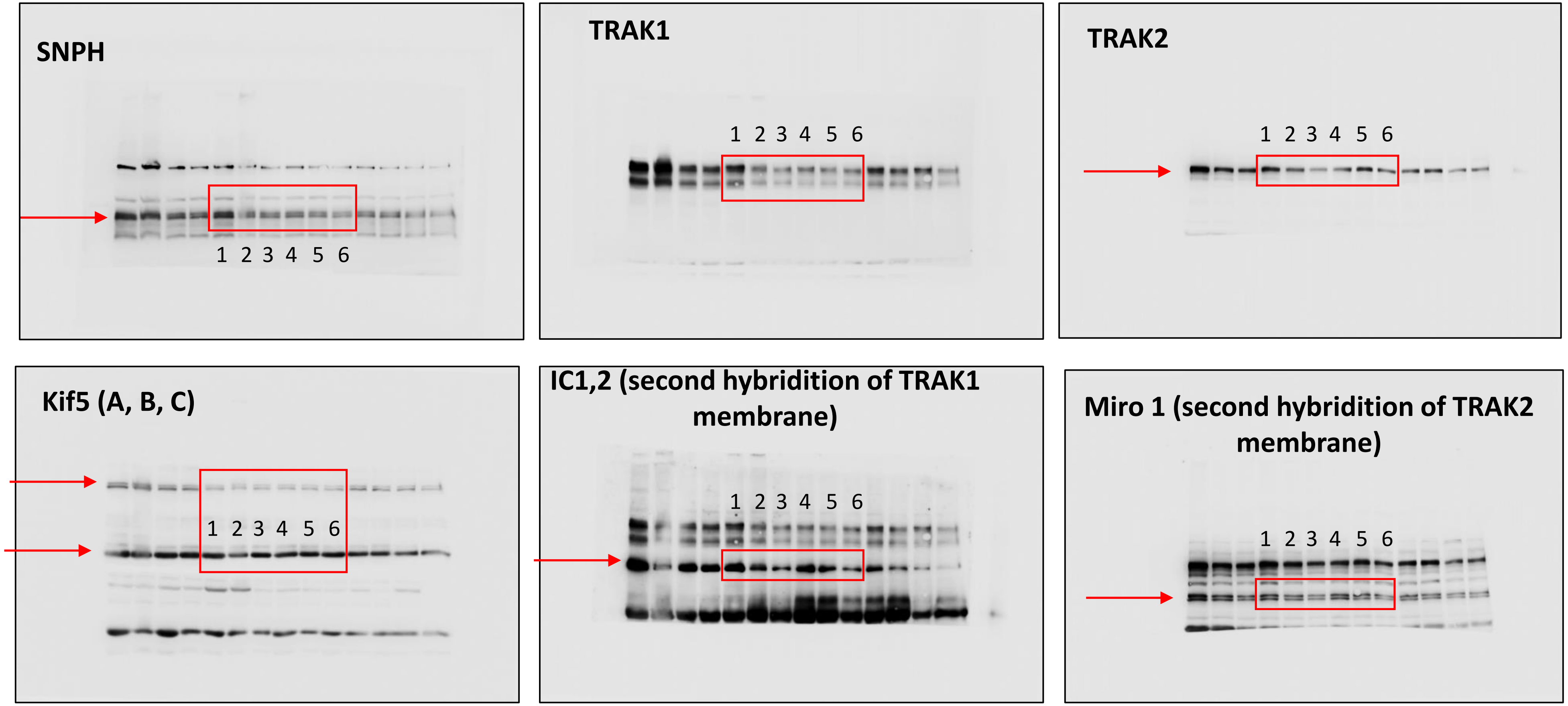

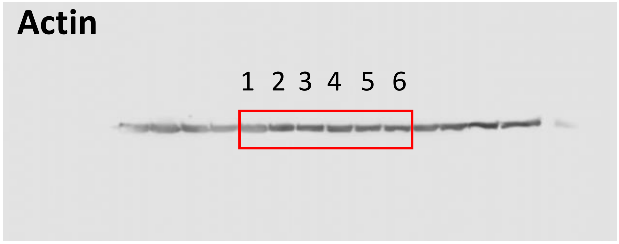

Related to Figure 7b

<u>13 months mice</u>

1, 2, 3 : WT

4, 5, 6 : 3xTgAD

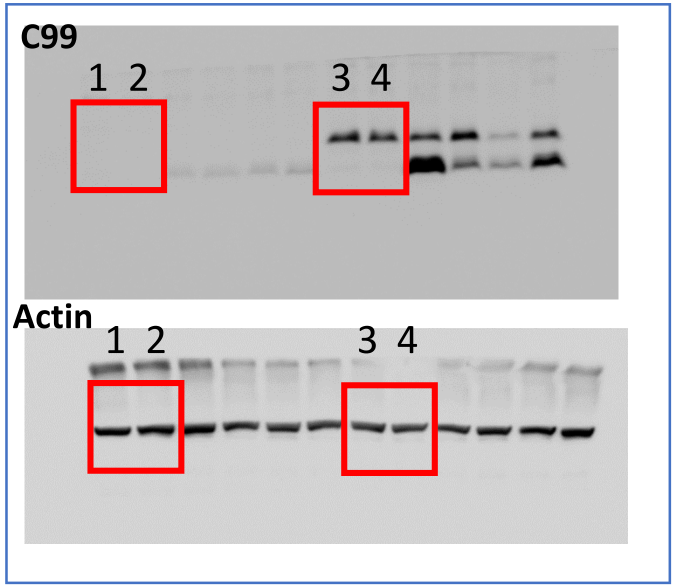

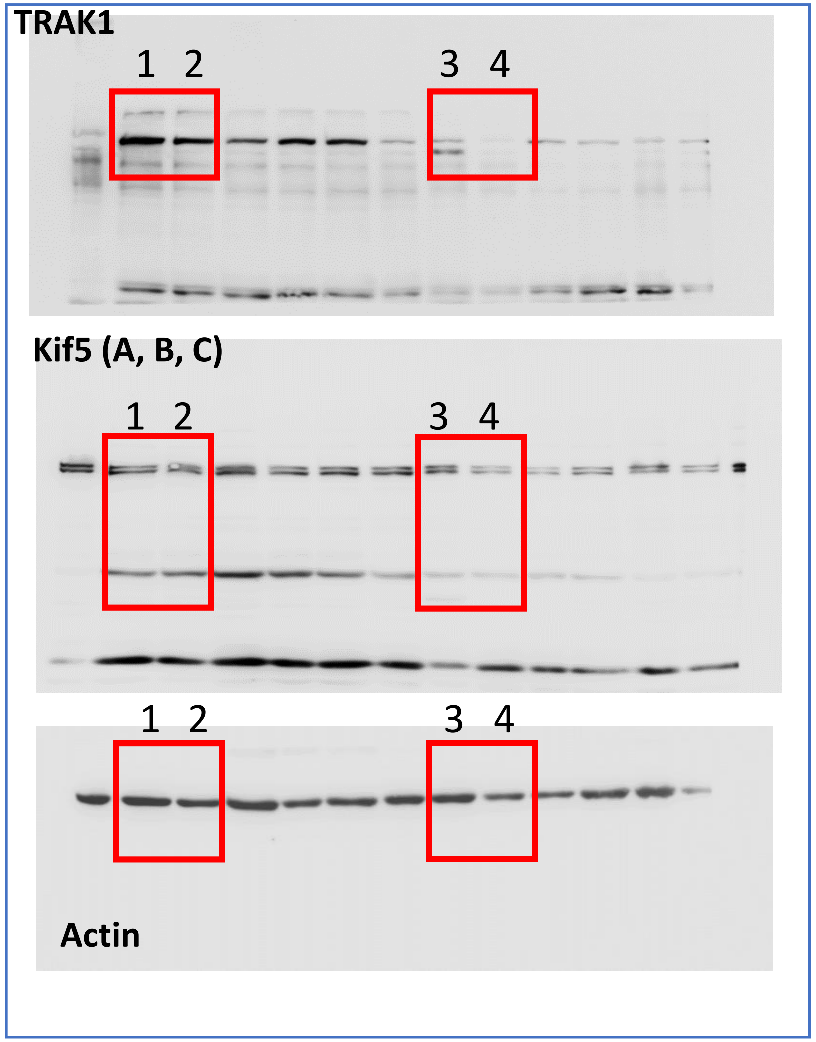

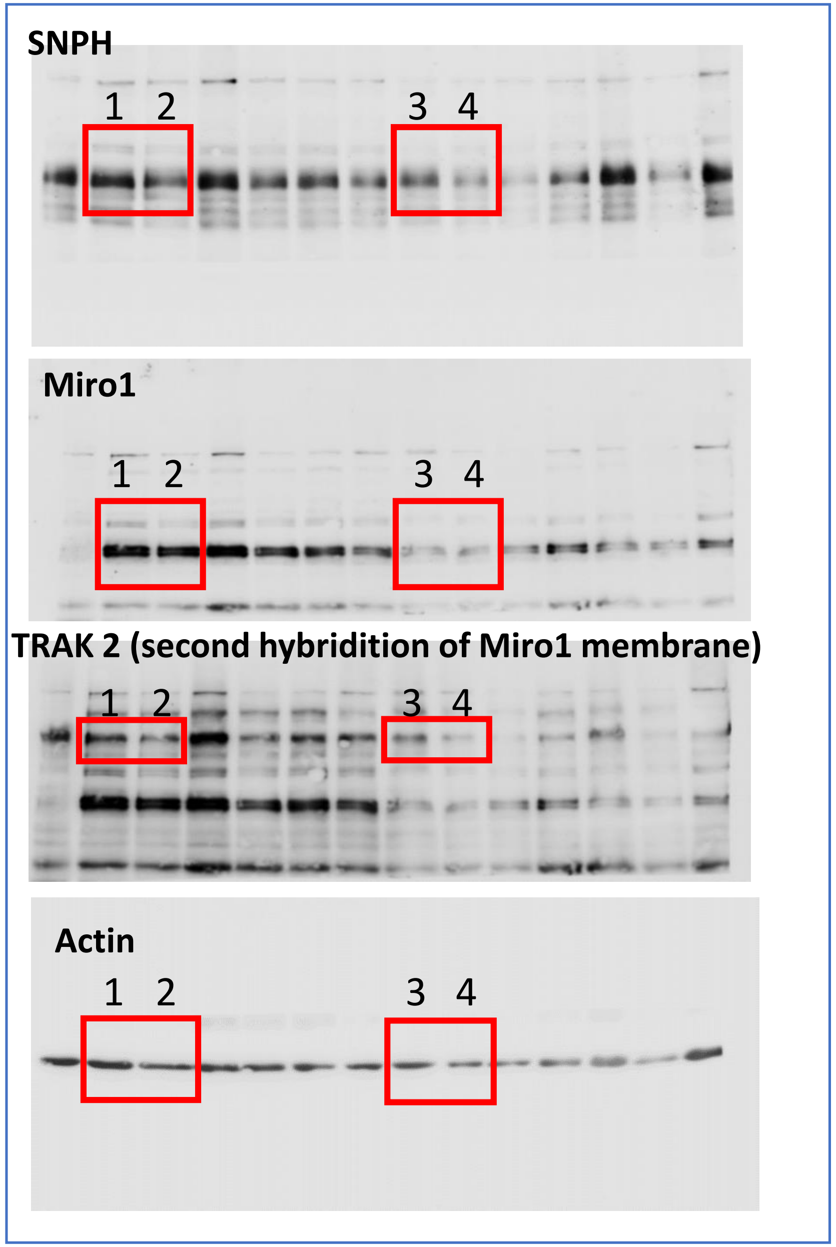

Mice

1 – 2 : AAV-Free

3 – 4 : AAV-C99

Related to Figure 8

Related to Figure 9

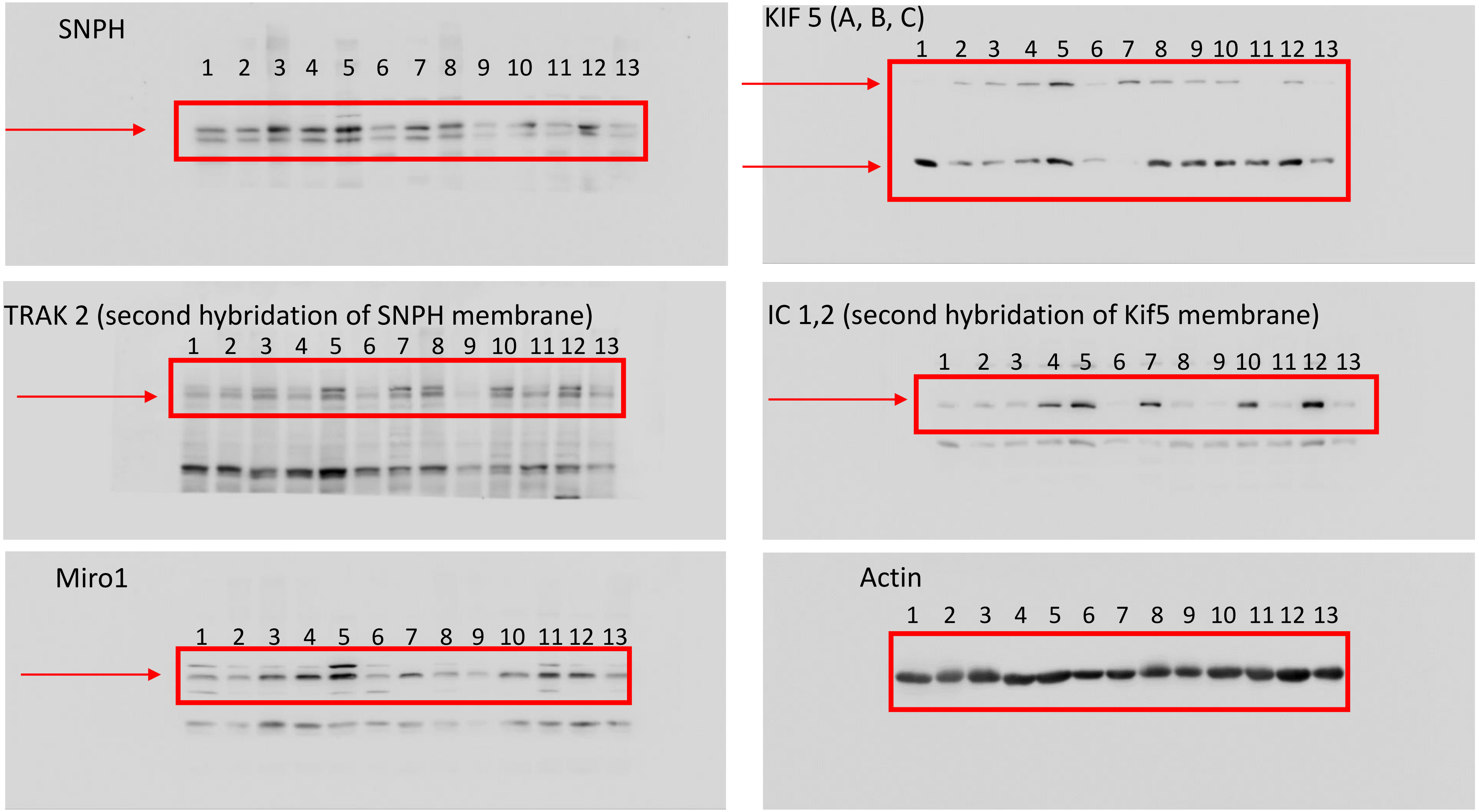

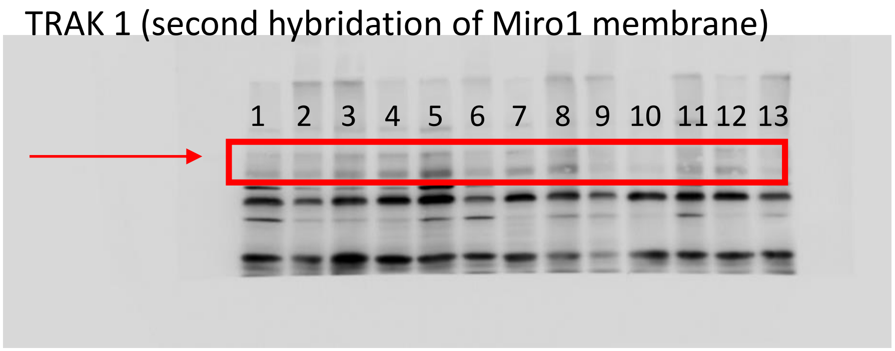

<u>**Human**</u>

1 – 3 : Controls
4 – 5 : Stage 2 SAD patients
6 – 7 : Stage 3 SAD patients
8 – 9 : Stage 4 SAD patients
10 – 11 : Stage 5 SAD patients
12 – 13 : Stage 6 SAD patients

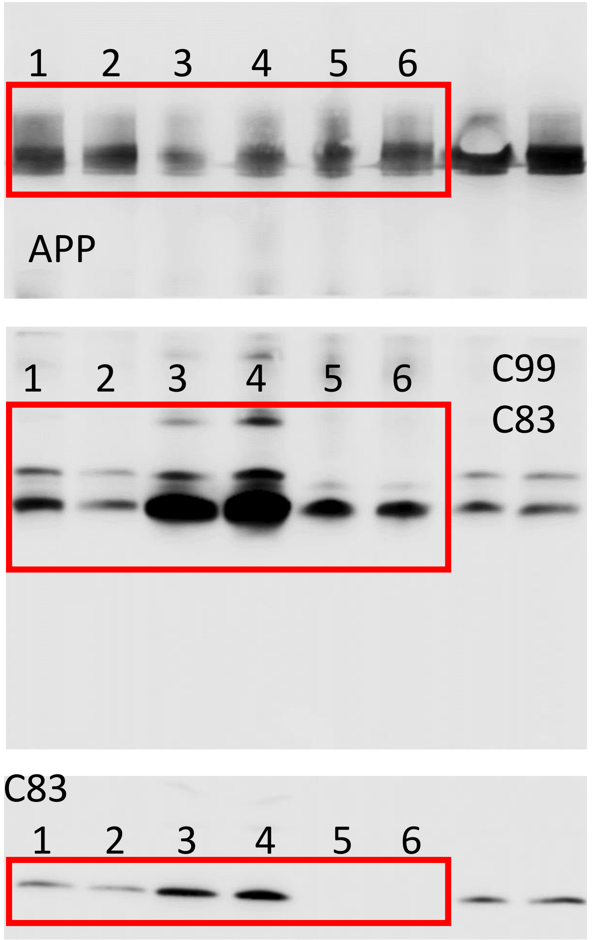

Related to Supplementary Figure 2a

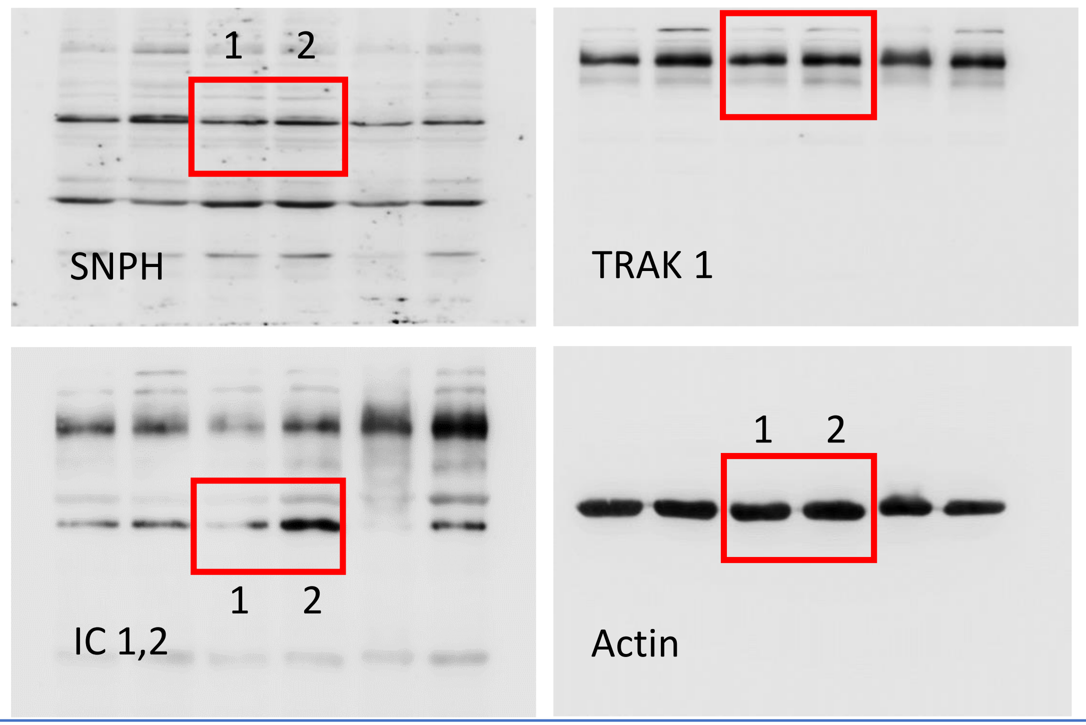

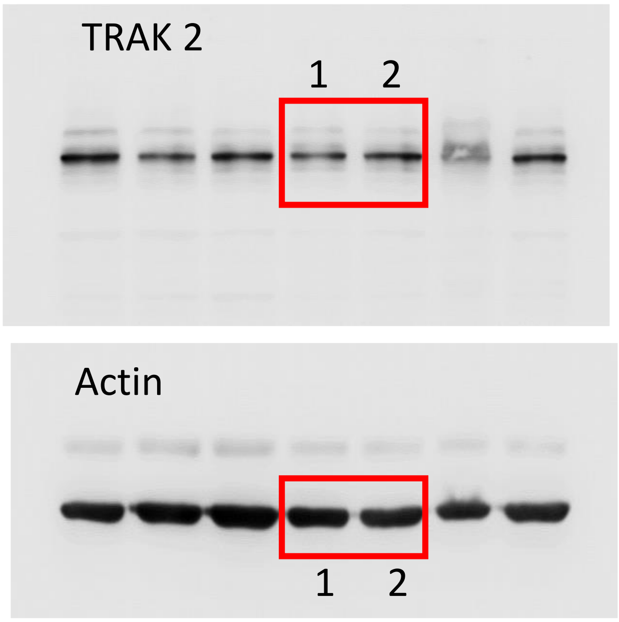

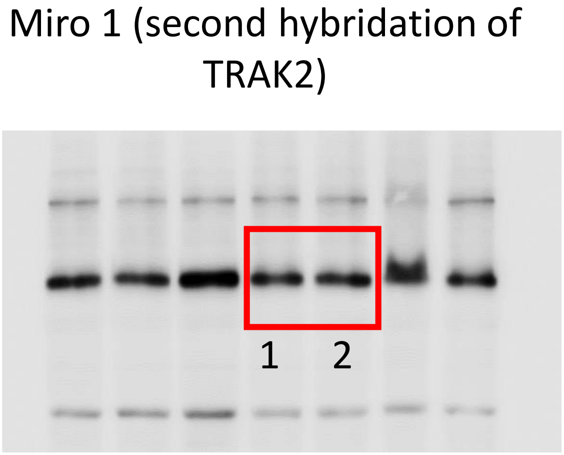

Related to Supplementary Figure 2b

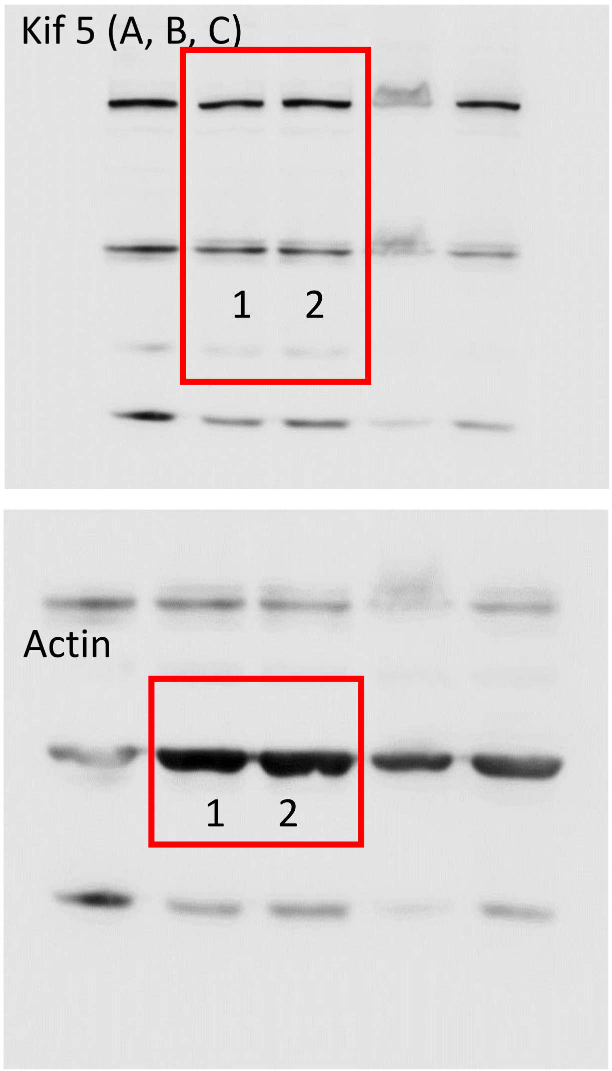

<u>**SH-SY5Y APPswe**</u>

1 – 2 : Veh

3 – 4 : γ-secretase inhibitor

5 – 6 : β-secretase inhibitor

<u>**SH-SY5Y APPswe**</u>

1 : Veh

2 : β-secretase inhibitor

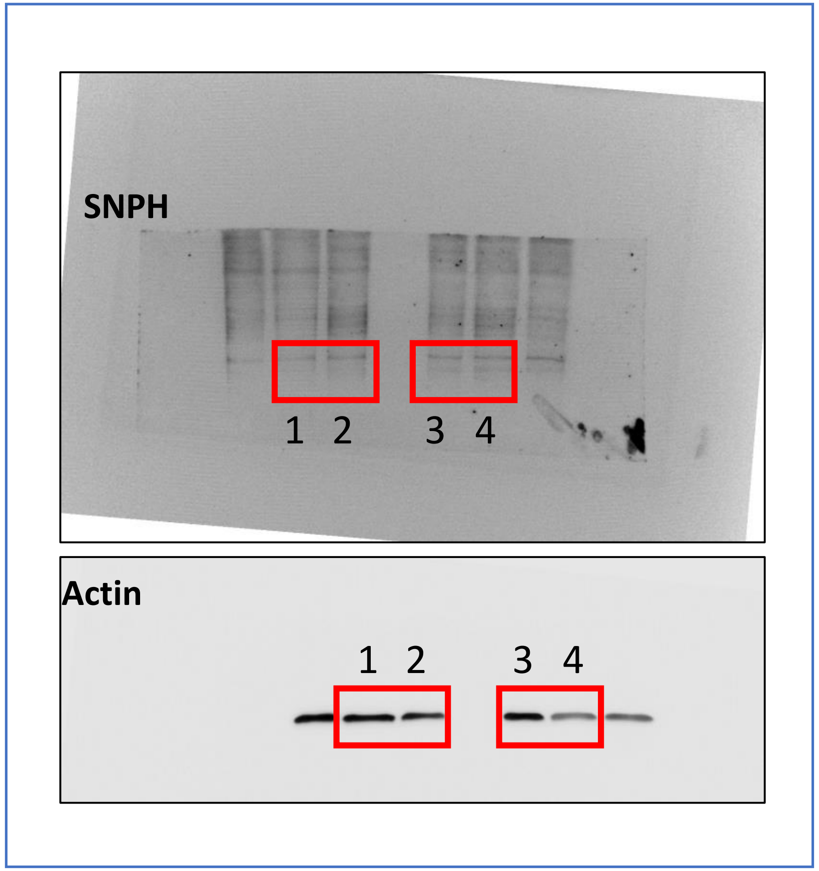

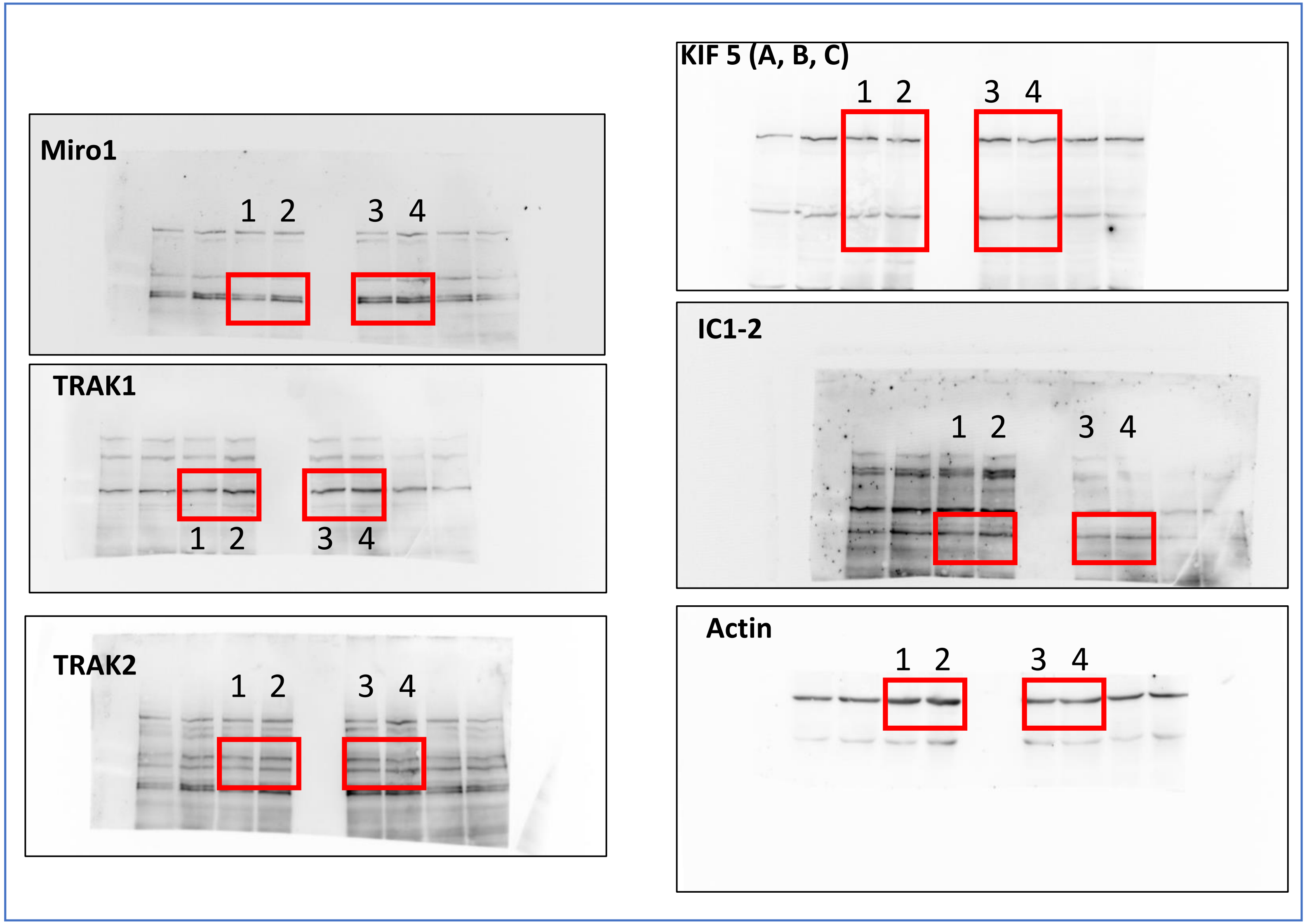

MEF APPKO

1 – 2 : Veh

3 – 4 : γ-secretase inhibitor

Related to Supplementary Figure 4

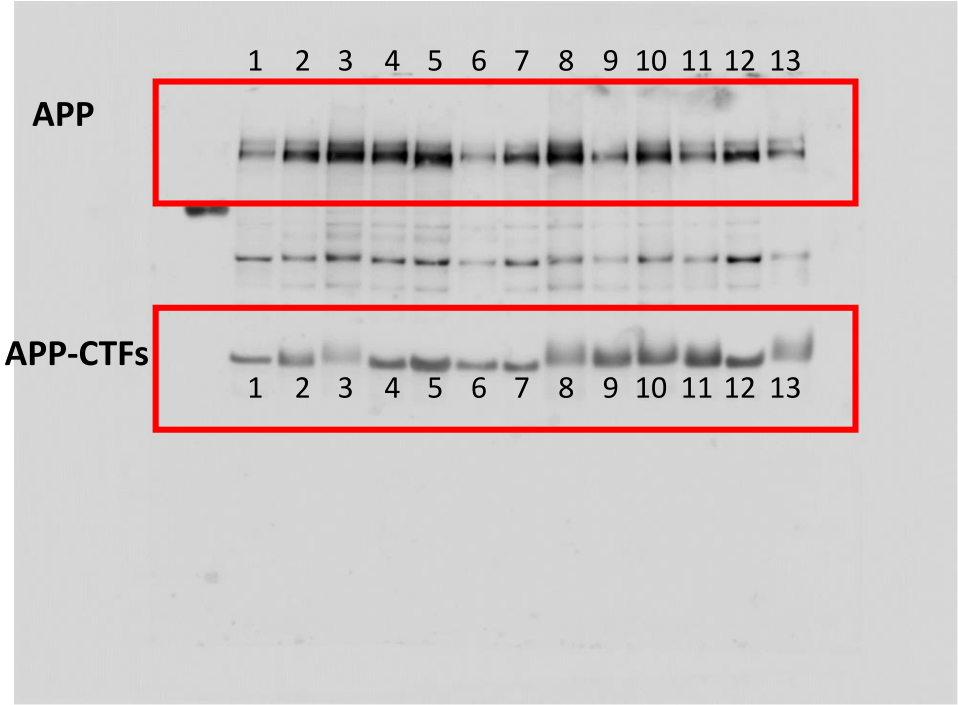

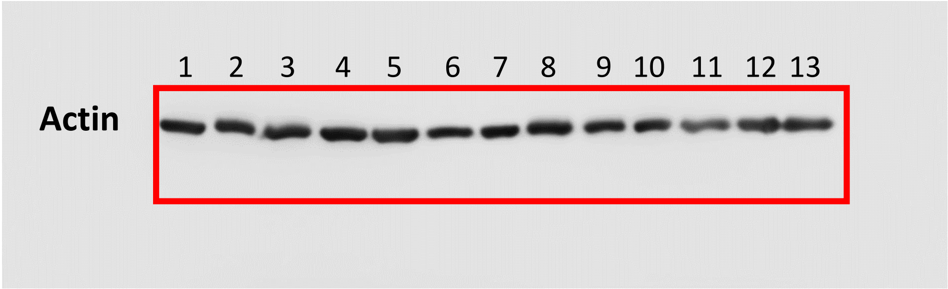

<u>Human</u>

1 – 3 : Controls
4 – 5 : Stage 2 SAD patients
6 – 7 : Stage 3 SAD patients
8 – 9 : Stage 4 SAD patients
10 – 11 : Stage 5 SAD patients
12 – 13 : Stage 6 SAD patients

Related to Supplementary Figure 8
